# Differential response of fire on the community dynamics of five insect taxa in a tropical mountaintop forest archipelago

**DOI:** 10.1002/ece3.10806

**Published:** 2023-12-06

**Authors:** Juliana Kuchenbecker, Flávio Camarota, Pedro Giovâni da Silva, Lucas Neves Perillo, Marina do Vale Beirão, Flávio Siqueira de Castro, Geraldo Wilson Fernandes, Mário Marcos do Espírito‐Santo, Natália Correia Santos, Iaciara Geórgia Silveira Cardoso, Frederico de Siqueira Neves

**Affiliations:** ^1^ Instituto de Ciências Biológicas, Programa de Pós‐Graduação em Ecologia, Conservação e Manejo da Vida Silvestre Universidade Federal de Minas Gerais Belo Horizonte Minas Gerais Brazil; ^2^ Departamento de Genética, Ecologia e Evolução, Laboratório de Ecologia Evolutiva e Biodiversidade Universidade Federal de Minas Gerais Belo Horizonte Minas Gerais Brazil; ^3^ Instituto de Ciências Biológicas, Departamento de Genética, Ecologia e Evolução, Laboratório de Ecologia de Insetos Universidade Federal de Minas Gerais Belo Horizonte Minas Gerais Brazil; ^4^ Instituto de Ciências Biológicas, Programa de Pós‐Graduação em Ecologia, Departamento de Ecologia Universidade de Brasília Brasília Distrito Federal Brazil; ^5^ Bocaina Biologia da Conservação Belo Horizonte Minas Gerais Brazil; ^6^ Departamento de Biologia Geral, Laboratório de Biologia da Conservação Universidade Estadual de Montes Claros Montes Claros Minas Gerais Brazil; ^7^ Programa de Pós‐Graduação em Biodiversidade e uso de Recursos Naturais Universidade Estadual de Montes Claros Montes Claros Minas Gerais Brazil

**Keywords:** dispersal capability, forest islands, insect conservation, temporal beta diversity, temporal patterns

## Abstract

The Earth's most diverse group of organisms is facing an imminent crisis, as recent investigations suggest a remarkable decline in insect diversity. Within this context, altimontane forest islands might emerge as important refuges holding an invaluable diversity of species that would be doomed to disappear. Here, we aimed to examine the impact of fire on the temporal variation of ant, bee, butterfly, dung beetle, and wasp communities in natural and highly threatened altimontane forest islands. We predicted that fire incidence would increase the natural variation in the structure of these insects' communities over time. Furthermore, we predicted that each taxon would respond accordingly to their ability to move between forest islands (i.e., vagility). We sampled these five bioindicator taxa in the rainy seasons of 2014, 2015, 2018, and 2020 within 14 forest islands in southeast Brazil. We assessed the incidence (presence/absence) of fire occurrence on each forest island toward the end of the dry season in each sampling year. We found an influence of fire incidence on the species composition changes over time (temporal *β*‐diversity) in the less vagile insect groups: ants, and dung beetles. Nevertheless, we found no influence of fire incidence on shifts in species composition of highly vagile insects: bees, butterflies, and wasps. Importantly, species turnover was the primary component of temporal *β*‐diversity driving the interannual variation of all insect taxa examined in this study. Our results highlight the distinct responses of more‐or‐less vagile insect groups to fire in forested ecosystems and shed light on the drivers of vulnerability and resilience of these groups to this critical anthropogenic pressure. By understanding and elucidating the intricate responses of distinct insect communities to global stressors, we can strengthen our capacity to predict future trends in biodiversity decline and provide valuable insights for conservation efforts and environmental management strategies.

## Graphical abstract

Fire incidence influences changes in species composition over time (temporal *β*‐diversity) in less vagile insect groups (ants and dung beetles). Species turnover is the primary component of temporal *β*‐diversity driving the interannual variation of all insect taxa examined in this study.

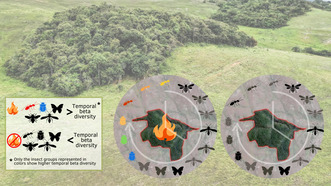

## 1 INTRODUCTION

The most diverse group of organisms on the planet is in trouble, with recent research suggesting that insect diversity is declining at an unprecedented rate (Cardoso et al., 2020; Eggleton, 2020; Sánchez‐Bayo & Wyckhuys, 2021). Among the insects facing population declines, some groups are of particular importance for the maintenance of ecosystem functioning and services, including bees (Potts et al., 2010), butterflies (Habel et al., 2016), hemipterans (Schuch et al., 2011), ants (Del Toro et al., 2012), and beetles (Dirzo et al., 2014). These insects are considered bioindicators, since they have the potential to reflect a given environmental status in relation to a reference ecosystem, revealing environmental changes in local environment scale (McGeoch, 2007). However, most studies on such insect groups are focused on their spatial distribution patterns related to land‐use changes (Schowalter, 2016), while temporal changes are still largely neglected (but see Donoso, 2017; Noriega et al., 2021; van Klink et al., 2020, 2021). Indeed, despite some important studies (e.g., Cappuccino & Price, 1995; Price & Hunter, 2015; Yamamura et al., 2006), the availability of time‐series data on insects is still limited and focused on a small range of taxa and a few types of human land use and habitats (Basset et al., 2023). Thus, there is a pressing need to better understand how the ongoing anthropogenic‐driven environmental changes act as drivers of alteration in the assembly of different insect communities over time. This is especially true due to the role of insects in the functioning of life on the planet and due to increasing evidence of their declining populations and extinctions (Hallmann et al., 2017; Sánchez‐Bayo & Wyckhuys, 2019; Seibold et al., 2019).

Amidst increasing anthropogenic disturbances, fire plays an important role in environments where it occurs naturally (fire‐prone ecosystems) and unnaturally (Barlow et al., 2020; França et al., 2021; Keeley & Pausas, 2019; Spies et al., 2012). Some of the most important fire‐prone ecosystems are comprised of open vegetation (e.g., grasslands and savannas), which are often permeated by non‐fire‐prone forests, mainly in the tropics (Dantas et al., 2013). In those specific fire‐prone ecosystems, wildfires can perpetuate or expand non‐forest vegetation through repeated fire episodes (Paritsis et al., 2015; Tepley et al., 2016) and, thus, can be beneficial for those insect species that are better adapted to live in non‐forest habitats. However, wildfires can have critically adverse effects on these fire‐prone ecosystem‐associated bioindicator insects (Batista et al., 2023; Kral et al., 2017), particularly under global warming (Jones et al., 2023; Keeley & Pausas, 2019). In particular, anthropogenic fires have been shown to reduce the abundance and diversity of most insects due to changes in vegetation structure and composition (Bieber et al., 2023). Such disturbance can cause drastic shifts in the dynamics of bioindicator insects and greatly influence their community composition (Vasconcelos et al., 2017). In general, fire induces both direct (e.g., death of adult individuals) (Kimuyu et al., 2014) and indirect insect mortality (e.g., nesting‐site destruction) (Rosa et al., 2021) attributable to habitat modifications and sets the stage for colonization and recolonization of neighboring unburned areas (Pausas, 2019; Pausas & Parr, 2018). However, despite some initial efforts, more studies on the effects of fire that take into account critical parameters of the insects, such as life‐history attributes and their ability to move between different areas, are needed (e.g., New, 2014; Swengel, 2001; Vidal‐Cordero et al., 2023). Such studies will help us to better understand how bioindicator insects re‐establish themselves in their habitats and to what extent these communities can resist increasing global changes.

Analysis of the reorganization of species composition over time (temporal *β*‐diversity) provides a powerful tool to detect the effects of fires on biological diversity (Magurran et al., 2019; Ulyshen et al., 2020). Studies conducted in tropical forests have shown that burned habitats had a lower replacement of hymenopteran species composition over time than unburned habitats (Tylianakis et al., 2005). Indeed, more pronounced reductions in site occupancy among bioindicators (e.g., butterflies and ants) have been reported in correlated habitats following fire incidence (Bishop et al., 2021; Carbone et al., 2019; Vasconcelos et al., 2017). However, in some cases, fire can also lead to an increase in site occupancy over time (Hawkins et al., 2015; Vidal‐Cordero et al., 2023). Overall, understanding these changes in species composition among taxonomic insect groups to anthropogenic disturbances will strengthen our capacity to predict future trends in biodiversity decline and guide conservation efforts at multiple scales (Chase et al., 2020; Socolar et al., 2016).

Within tropical ecosystems, mountainous regions exhibit heightened vulnerability to drivers of global change, with land‐use alterations standing out as a particularly influential factor (Colwell et al., 2008; Hoorn et al., 2018; Rumpf et al., 2018). In some of those mountains, we can find a system comprised of altimontane forests (fire‐sensitive) surrounded by a fire‐prone grassland matrix (Alvarado et al., 2017; Coelho et al., 2018). These forest archipelagos form a distinctly evergreen ecosystem, confined to a narrow altitudinal range (Coelho et al., 2016; Foster, 2001; Richter et al., 2008). This environment is marked by restricted features (e.g., the intersection of fog incidence, soil composition, and mountain slopes) that underscore not only the ecosystem's susceptibility to ongoing anthropogenic changes (Hamilton et al., 1995), but also the challenges associated with safeguarding it from such changes (Vuille et al., 2003). Given the environmental variability typical of these forest archipelagos, studies encompassing different time series are critical to understanding temporal dynamics among insect groups, as they allow us to highlight the main drivers of directional changes in their composition (Chase et al., 2020). Importantly, these forest–island ecosystems may serve as important refuges holding an invaluable diversity of species that would be doomed to disappear (Janzen & Hallwachs, 2019; Neves, Antoniazzi, et al., 2021; Noriega & Realpe, 2018).

In this study, we aimed to examine the influence of fire incidence on the temporal variation of ant, bee, butterfly, dung beetle, and wasp communities in natural and highly threatened altimontane forest islands. All these insect groups encompass a range of different natural history attributes, such as body size, foraging patterns, and dispersal ability. Notably, there are striking differences in the dispersal abilities of these groups, ranging from low‐vagility organisms (e.g., ants) to high‐vagility ones (e.g., butterflies) (see Renault, 2020 for a review). Given that anthropogenic disturbances, such as intensive fires, are often associated with significant changes in insect communities even after a few years (Paolucci et al., 2016; Rosa et al., 2021), we hypothesized that (i) the incidence of fire on forest islands will cause a greater variation, compared with undisturbed habitats, in the structure (species richness and composition) of these communities over time, mainly in groups of lower dispersal capability. We also hypothesized that (ii) turnover will be the main component responsible for the temporal variation of all insect taxa presented in this study. This second hypothesis follows from the recognition that these distinct insect groups may exhibit varying mobility rates among forest islands (Camarota et al., 2023), reflecting regionally organized metacommunities and implying a change in species composition over time (da Silva et al., 2019; da Silva, Camarota, et al., 2023). Additionally, species turnover among sites tends to be more important than nestedness in determining insect community organization within a region, especially in insular habitats (see Brant et al., 2021; da Silva et al., 2019; Kuchenbecker et al., 2021; Perillo et al., 2020 for the same system).

## 2 MATERIALS AND METHODS

### 2.1 Study area

**FIGURE 1:**
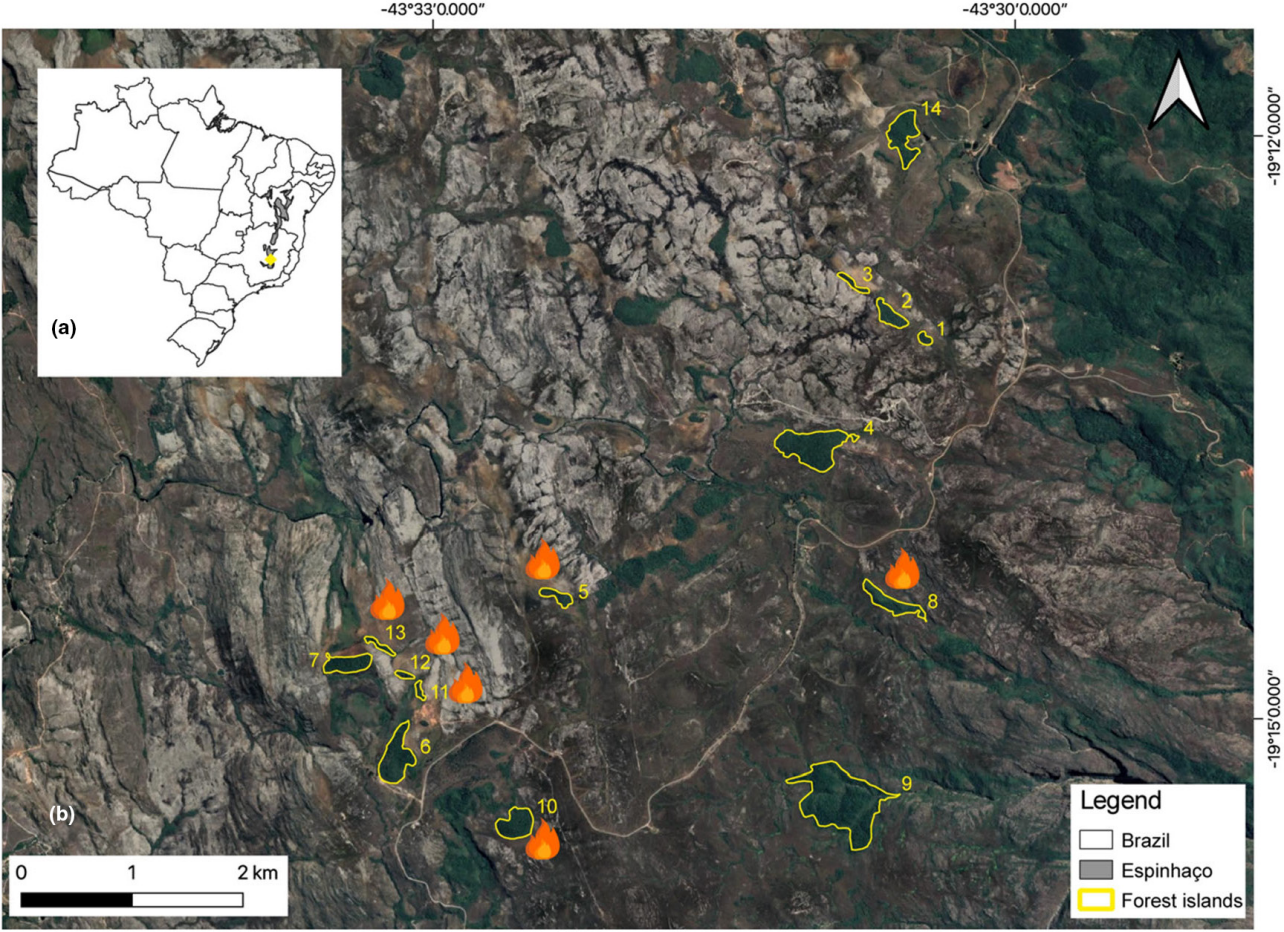
Map showing (a) Espinhaço Range in Brazil and (b) location and fire records (fire symbol) of the 14 natural forest islands (yellow shapes) where we sampled ant, bee, butterfly, dung beetle, and wasp communities within a *campo* r*upestre* matrix of the *Serra do Cipó*, southern portion of the Espinhaço Range Biosphere Reserve, Minas Gerais, Brazil.

This study was conducted in a natural forest archipelago (i.e., natural forest patches immersed in grassland matrix) located in the southern portion of the Espinhaço Range Biosphere Reserve at the *Serra do Cipó* mountain complex, Brazil (19°14′19″ S, 43°31′35″ W; Figure 1). The Espinhaço Range, South America's second largest mountain complex (after the Andes), holds significant importance in terms of biodiversity and endemism (see Fernandes, 2016a; Fernandes et al., 2020 for reviews). Spanning over 1200 km from southeast to northeast Brazil, the width of the range seldom exceeds 100 km in an east–west direction (Fernandes, 2016a, 2016b; Silveira et al., 2016). Within the Espinhaço Range, three Brazilian biomes can be found: the Caatinga, a semi‐arid dry forest to the north; the Cerrado, a Brazilian savanna to the west; and the Atlantic Forest, a tropical rainforest to the east (Fernandes, 2016b; Silveira et al., 2016). The latter two biomes are renowned as biodiversity conservation hotspots (Myers et al., 2000). The Espinhaço elevation range varies from 650 to 2072 m a.s.l. Particularly, above 900 m a.s.l. we can find a grassy ecosystem matrix known as *campo rupestre*, an ancient, climatically buffered, and diverse ecosystem that is considered a Brazilian biodiversity hotspot and hosts various endangered and endemic species (Fernandes et al., 2020; Hopper et al., 2021). This ecosystem comprises highly heterogeneous herbaceous/shrubby vegetation, consisting of rocky outcrops scattered amidst sandy and stony grasslands, seasonal wet grasslands, and occasional forest islands (Fernandes, 2016a). The specific edaphic‐climatic characteristics, such as erosion valleys lacking boulders within the *campo rupestre*, enable the presence of those relictual forest islands (Coelho et al., 2016) commonly found on mountaintops above 1200 m a.s.l. These forest islands exhibit a floristic composition similar to the semi‐deciduous forests associated with the Atlantic Forest domain (Coelho et al., 2016, 2018) and are part of a natural forest archipelago (i.e., shaped without anthropic intervention). Importantly, these islands represent a distinctive feature of the transitional zone between the Cerrado and the Atlantic Forest biomes across the Espinhaço Range, offering valuable opportunities for testing ecological and evolutionary hypotheses in natural systems (Coelho et al., 2018).

The elevation of the sampled forest archipelago ranges between 1230 and 1331 m a.s.l., where the regional climate is Cwb (dry‐winter subtropical highland climate) according to Köppen's classification (Alvares et al., 2013), with dry winters and rainy summers. The annual rainfall ranges from 700 to 1500 mm, with higher values in summer (167.3 mm/month; December to March) than in winter (13.4 mm/month; June to September). The monthly mean temperature ranges between 13.9 and 21.9°C, with higher values in summer (20.6 ± 0.3°C) than in winter (17.1 ± 0.8°C). The forest islands' average air humidity is 79.8 ± 2.4% in summer and 75.3 ± 2.5% in winter (data from a meteorological station Onset HOBO U30 data logger, which is installed at 1300 m a.s.l. within the study region).

### 2.2 Sampling design

We sampled five taxa of bioindicator insects (i.e., ants, bees, butterflies, dung beetles, and wasps) (see Appendix S1 for detailed information) within 14 forest islands. These areas belong to the permanent plots of the Long‐Term Ecological Research Project *Campos Rupestres da Serra do Cipó* (PELD‐CRSC) (Figure 1). The chosen forest islands differed in size, distances among each other and from continuous forest areas (i.e., possible source of forest‐like species) (see Table S1; Figure S1). In the central region of each forest island, a 20 × 50 m plot was established, where we performed a four‐year sampling in the rainy seasons of 2014, 2015, 2018, and 2020. In each sampling period, we used non‐baited pitfall traps for the ant sampling, which consisted of a plastic container (9 cm deep × 15 cm diameter), with 250 mL of a salt and detergent solution (5% each), and a rain protection placed in the corners and in the center of the plot (*n* = 5) (Brant et al., 2021). Similar traps were used to sample dung beetles, but those traps were baited with 25 g of human feces and placed only in the corners of the plot (*n* = 4). Each pitfall trap (ant and dung beetle) was left in the field for 48 h per sampling (da Silva et al., 2019). For butterflies, we used Van‐Someren Rydon traps baited with a mix of fermented banana and sugar cane juice placed in the four corners of the plot. Each trap was left in the field for 5 days and was inspected daily (Pereira et al., 2017). Finally, for bees and wasps, we used two Malaise traps (one Malaise trap in the ground with approximate dimensions of 210 cm height × 210 cm width × 210 cm length—see Souza et al., 2015, and one canopy Malaise Window trap with approximate dimensions of 280 cm height × 100 cm width × 250 cm length) in the center of the plot, which remained in the field for 7 days (Perillo et al., 2020).

Due to logistical constraints and resource shortages in 2014 and 2015, we were unable to sample butterflies in three of the 14 forest islands during the rainy season of these years. Thus, only comparisons between 2018 and 2020 were based on 14 forest islands for butterflies; all comparisons with 2014 or 2015 were based on 11 forest islands. Each specimen was identified to the lowest possible taxonomic level using identification guides (Baccaro et al., 2015; Canals, 2003; DeVries, 1987; Fernández & Sharkey, 2006; Silveira et al., 2002; Vaz‐de‐Mello et al., 2011) and consultation with taxonomic experts (see Acknowledgments). The sampled material was deposited in the *Centro de Coleções Taxonômicas of the Instituto de Ciências Biológicas* at the Universidade Federal de Minas Gerais (bees and wasps), in the *Zoological Collection of the Universidade Federal do Paraná* (ants), in the *Universidade Estadual de Campinas* (butterflies), and in the *Universidade Federal de Mato Grosso* (dung beetles). All necessary sampling permits were issued by *Instituto Chico Mendes de Conservação da Biodiversidade* (SISBIO #57764‐1).

During each field campaign within the sampling period (2014–2020), we assessed the frequency of fire occurrence within each plot of each forest island. Toward the end of the dry season (mid‐October) in each year, we performed detailed observations by examining the presence or absence of remnants of recently burned vegetation inside the plots. Additionally, we gathered information on recent fire events from local landowners (Figure 1; Table S1). It is worth noting that not every fire that affected the forest islands reached the analyzed plots, and we only assessed the fire events that actually reached the plots.

### 2.3 Statistical analyses

We constructed rarefaction and extrapolation curves using the R package “iNEXT,” available at https://chao.shinyapps.io/iNEXTOnline/, to calculate values of species richness to a similar sample coverage (Chao et al., 2014; Hsieh et al., 2016). We accounted for the frequency of occurrence (i.e., presence/ absence) for ants and for every individual collected for the other four groups of insects (bees, butterflies, beetles, and wasps). Thus, we estimated the sample completeness for each group. For extrapolation curves, the number of individuals was twice the actual reference size (Chao et al., 2014). We then built rarefaction curves with different sampling intensities, with these curves representing the gamma diversity of each sampling year (2014, 2015, 2018, and 2020) per insect group. We used the sample‐based approach (i.e., the 14 forest islands) for ants and the individual‐based approach for bees, butterflies, dung beetles, and wasps. The species richness of each insect group was considered as a dependent variable. Thus, we were able to obtain information about the completeness of each insect group species in relation to each sampling unit/number of individuals.

We assessed the temporal variation of each taxonomic group composition (temporal *β*‐diversity) using the Sørensen dissimilarity index in a multiple‐time approach (see Baselga, 2010; da Silva, Salomão, et al., 2023; Nunes et al., 2020). Then, we partitioned total temporal *β*‐diversity among the forest islands into turnover and nestedness‐related components using the “beta.multi” function in the “betapart” R package (Baselga & Orme, 2012). Data were aggregated by sampling years for partitioning *β*‐diversity over sampling dates. The function “beta‐multi” computes multiple‐site dissimilarities *β*_SOR_, *β*_SIM_, and *β*_NES_, respectively, to represent total *β*‐diversity, turnover‐resultant component, and nestedness‐resultant component (Baselga, 2010). We used the results of this calculation to determine whether species turnover or the nestedness‐resultant components had the greatest influence on the general pattern of temporal *β*‐diversity (Baselga, 2010; da Silva, Salomão, et al., 2023; Nunes et al., 2020).

To test for the effects of fire on the temporal variation of all the insect group communities, we used separate generalized linear models (GLM's). Since we have species richness for each collection year, for each forest island and each insect group, we calculated the coefficient of variation (richness variation; or Δ) by dividing the standard deviation by the mean of these richness values. In our GLM's, the richness variation and temporal *β*‐diversity were the dependent variables, and fire was the independent variable in different models for each evaluated taxonomic group. The R function “Anova” was used to deem variables significant or not. All models were subjected to residual analyses to check for model fit and error structure suitability (Crawley, 2013). All statistical analyses were performed using R software v.4.0. (R Core Team, 2023).

## 3 RESULTS

### 3.1 General patterns

Between 2014 and 2020, we sampled 377 insect species belonging to the studied bioindicator taxa: 114 ants, 35 bees, 42 butterflies, 55 dung beetles, and 131 wasps (Table S2). The accumulated insect species richness per forest island ranged between 71 and 126 species (average ± sd: 103.1 ± 13.8 species), with 33 and 46 species of ants (39.8 ± 3.8), one and 15 species of bees (5.1 ± 3.9), one and 16 species of butterflies (8.3 ± 4.8), 13 and 22 species of dung beetles (16.4 ± 2.9), and 16 and 49 species of wasps (33.6 ± 9.0). The ants *Acromyrmex subterraneus* (Forel, 1893), *Camponotus lespesii* Forel, 1886, *Camponotus rufipes* (Fabricius, 1775), *Holcoponera striatula* (Mayr, 1884), *Pachycondyla striata* Smith, 1858, *Pheidole jelskii* Mayr, 1884, *Pheidole oxyops* Forel, 1908, *Pheidole* sp. 8, *Solenopsis* sp. 1, the dung beetles *Uroxys* sp. 1 and *Uroxys* sp. 2, and the wasps *Agelaia multipicta* (Haliday, 1836) and *Polybia fastidiosuscula* Saussure, 1854 (Vespidae), and *Pristocerinae* sp. 5 (Bethylidae) were the most widespread species, occurring in all forest islands over the years. The most widespread bee was *Melipona* (*Melipona*) *quadrifasciata* Lepeletier, 1836, occurring in 10 forest islands, while the butterfly *Yphthimoides straminea* (Butler, 1867) was the most widespread butterfly, occurring in 11 forest islands.

The sampling effort was sufficient to characterize the insect community, with the rarefaction‐extrapolation accumulation curves presenting a greater sampling coverage for wasps, followed by ants, dung beetles, bees, and butterflies, respectively (Figure S2). We noticed that the observed and estimated wasp's species richness ranged between 131 and 201 species, respectively, corresponding to 98.1% of the sampling coverage value over the years. For the ants, we found observed and estimated species richness ranging between 114 and 143 species, respectively (97% of the sampling coverage). For the dung beetles, the observed and estimated species richness ranged between 55 and 73 species, respectively (99.6% of the sampling coverage). Bees had their observed and estimated species richness ranging between 35 and 83 species, respectively (87.3% of the sampling coverage). Finally, the observed and estimated butterfly species richness ranged between 42 and 50 species (98.1% of the sampling coverage).

### 3.2 Effects of fire incidence on temporal insect diversity variation

**FIGURE 2:**
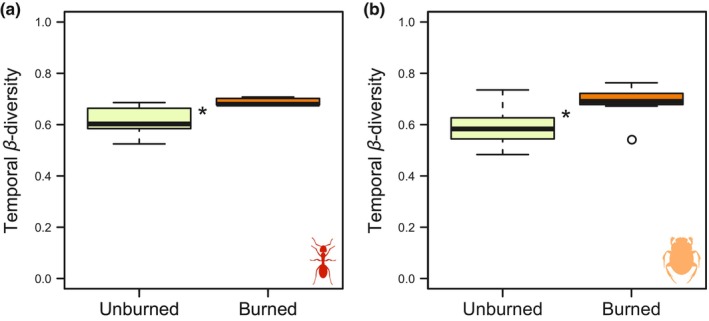
Boxplots of (a) ants and (b) dung beetles temporal *β*‐diversity in forest islands at the Espinhaço Range, southeastern Brazil, considering fire incidence and absence.

Fire incidence had no influence on any of the dependent variables (i.e., richness variation and temporal *β*‐diversity) for bees, butterflies, and wasps (Table 1). Similarly, the richness variation of ants and dung beetles was not influenced by fire incidence. Only the temporal *β*‐diversity of ants and dung beetles showed fire‐mediated effects (Table 1), with burned forest islands presenting higher values of temporal *β*‐diversity for ants (Table 1, Figure 2a) and dung beetles (Table 1, Figure 2b).

**TABLE 1:** Generalized linear models showing the effects of fire occurrence on the variation of insect species richness and temporal beta diversity of ants, bees, butterflies, dung beetles, and wasps. *p*‐values <.05 are in bold.

Taxa	Dependent variables	Independent variable	df	*F*‐value	Pr (>*F*)
Ants	Richness variation	Fire	1, 12	0.16	.69
	Temporal *β*‐diversity	Fire	1, 12	6.04	**.03**
Bees	Richness variation	Fire	1, 12	1.21	.29
	Temporal *β*‐diversity	Fire	1, 12	3.92	.07
Butterflies	Richness variation	Fire	1, 12	0.68	.47
	Temporal *β*‐diversity	Fire	1, 12	1.48	.27
Dung beetles	Richness variation	Fire	1, 12	0.37	.56
	Temporal *β*‐diversity	Fire	1, 12	5.05	**.04**
Wasps	Richness variation	Fire	1, 12	0.76	.40
	Temporal *β*‐diversity	Fire	1, 12	0.75	.40

### 3.3 Partition of diversity

**FIGURE 3:**
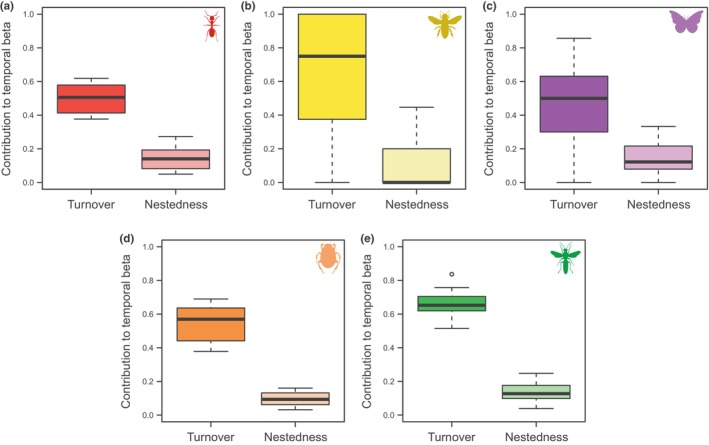
Boxplots of turnover and nestedness contribution to temporal *β*‐diversity of (a) ants, (b) bees, (c) butterflies, (d) dung beetles, and (e) wasps in forest islands at the Espinhaço Range, southeastern Brazil.

When we partitioned temporal *β*‐diversity into its turnover and nestedness components, we verified that temporal *β*‐diversity was mostly represented by the turnover (*β*_SIM_) component for all insect groups (Figure 3). This component explained more than 75% of the total *β*‐diversity for all groups (Table 2).

**TABLE 2:** Temporal *β*‐diversity using the Sørensen index for all sampled insect groups and the relative importance (%) of turnover (*β*_SIM_/*β*_SOR_) and nestedness (*β*_NES_/*β*_SOR_) for each one of them.

Insect group	Temporal *β*‐diversity	Turnover	Turnover	Nestedness	Nestedness
	Mean ± SD	Mean ± SD	(%)	Mean ± SD	(%)
Ants	0.65 ± 0.06	0.50 ± 0.08	77	0.15 ± 0.08	23
Bees	0.88 ± 0.14	0.66 ± 0.35	75	0.09 ± 0.13	10
Butterflies	0.59 ± 0.23	0.45 ± 0.27	76	0.15 ± 0.09	25
Dung beetles	0.64 ± 0.09	0.54 ± 0.10	84	0.10 ± 0.04	16
Wasps	0.80 ± 0.04	0.66 ± 0.08	82	0.14 ± 0.05	18

## 4 DISCUSSION

In this study, we evaluated the impact of fire incidence on the temporal variation in the communities of five bioindicator insect group: ants, bees, butterflies, dung beetles, and wasps. We highlight a significant influence of fire on the species composition changes in ants and dung beetles over time. Notably, we observed that these low‐vagility organisms (ants and dung beetles) showed a greater susceptibility to the reorganization of species composition over time in the face of this anthropogenic disturbance, in comparison to their high‐vagility counterparts (bees, butterflies, and wasps). Our results underscore the potential importance of different factors in shaping the temporal patterns of insect species distribution in forest islands. Among these factors, the role of dispersal ability stands out as particularly noteworthy. Furthermore, our study provides insights into how the life history attributes and the degree of habitat specialization within the five bioindicator insect groups could potentially contribute to the observed changes in species composition over time.

Ecological processes, such as dispersal, can change variation in species compositions over time irrespective of environmental conditions (Fukami, 2015). In fact, dispersal is a crucial process for species survival within forest‐grassland mosaics (Fahrig, 2003), exhibiting variable success and rates among individuals and species, thereby exerting influence over metacommunity dynamics (Leibold et al., 2004; Renault, 2020; van der Plas et al., 2023). Here, we found (i) no discernible impact of fire incidence on the richness variation and temporal *β*‐diversity for bees, butterflies, and wasps. These findings are consistent with other studies that have reported little or no short‐term impacts of fire on arthropods (Andersen & Müller, 2000; Hanula et al., 2012). However, we found significant effects of fire on the temporal *β*‐diversity for ants and dung beetles, which partially corroborates our first hypothesis, since these were the studied groups with the lowest dispersal ability. Concordantly, differences in dispersal capability among species groups can have important effects on their temporal patterns (da Silva, Camarota, et al., 2023), particularly in tropical altimontane forest‐grassland mosaics. In such mosaics, fires can reshape the entire structure of the landscape, generating insect communities that are increasingly disjointed in space and time (Camarota et al., 2023; Neves, da Silva, et al., 2021). This is especially true for ground‐dwelling insects such as most ants and dung beetles, which are predicted to be most affected by fire‐driven habitat loss (Koltz et al., 2018; Warren et al., 2001).

The differences related to the dispersal capability of the different insect groups are not the only factor to be considered, and in some cases, the dispersal ability can act together with aspects of their life histories. Thus, to fully understand the temporal patterns observed, we must consider both the dispersal and the life history attributes of the organisms. For example, due to their low vagility, most ants and many dung beetle species are highly dependent on local resources and conditions to forage (Camarota et al., 2016; da Silva & Hernández, 2015), with a minor chance to use other habitats (Brant et al., 2021). While ants can temporarily move their colonies during disturbances, this is only possible in adjacent habitats (e.g., Arruda et al., 2022), which are not separated by, for instance, a distinct vegetation matrix, such as our focal forest islands. In addition, while most ants and dung beetles' nest in the ground layer, which is relatively safe from fire (Cane & Neff, 2011), many other ant species nests in the litter or in the vegetation (Priest et al., 2021), and their nest sites can be completely destroyed after a fire (Arruda et al., 2020; Rosa et al., 2021). Consequently, when confronted with changes in resource availability and in habitat conditions caused by anthropic fires, these less mobile insect groups have limited possibilities to persevere, and many species face local extinctions (Leibold & Chase, 2018). In addition to the reliance on local resources, most ant species are only able to disperse during the mating season (winged queens and males) (Hakala et al., 2019). The same cannot be applied to the other species with greater mobility (i.e., bees, wasps, and butterflies), in which fire had no effect on changing their species composition over time. In this regard, wasp and bee species are held as good fliers (Compton et al., 2000; Venkateswaran et al., 2017). Similarly, most butterfly species also have several types of movement, ranging from short distances for feeding and breeding over a few hours or days to seasonal, long‐distance migrations (Bhaumik & Kunte, 2020). Therefore, it is reasonable that both species richness and species composition of these insect groups do not vary significantly over time in response to anthropic fires in the study system. Nevertheless, a potentially dramatic increase in fire frequency may promote biotic homogenization among forest islands, reducing changes in species composition and favoring colonization by habitat generalist species.

Consistent with our expectations, we also found that (ii) species turnover was the primary component driving the interannual variation of all insect taxa examined in this study. Recent studies carried out in the same forest archipelago examining separately each of the taxa in this study (see Brant et al., 2021 for ants; da Silva et al., 2019 for dung beetles; Pereira et al., 2017 for butterflies; Perillo et al., 2020 for wasps and bees), identified high seasonal species turnover rates among forest islands, pointing out the significance of each forest island in maintaining the structure and conserving the communities associated with the entire archipelago. This turnover of species, probably by a similar number of species lost, can potentially explain the lack of effect of fire on species richness variation as well.

After the occurrence of fire on forest islands, there is a considerable loss of area and forest cover (Coelho et al., 2018). During the regeneration process, habitat generalist species and those associated with the *campo rupestre* can easily be sampled in the area formerly comprised by the forest island, where our long‐term sampling plot is set up (Neves, Antoniazzi, et al., 2021). In this sense, the replacement of forest island‐associated species by habitat generalist species or those associated with open environments can potentially explain the high compositional change represented mainly by the turnover that we found in the forest islands. For example, da Silva et al. (2019) found that habitat generalist species of dung beetles are richer and more abundant on forest islands with lower canopy cover during the rainy season (summer). The variation in canopy cover is dependent on the tree species present there (Coelho et al., 2018), but can be strongly influenced by the occurrence of fire. For example, the dung beetle *Sulcophanaeus menelas* (Castelnau, 1840), commonly sampled in the *campo rupestre* between 900 and 1400 m (Nunes et al., 2016, 2018), began to be sampled on forest islands after the occurrence of fire. The same occurred with ants, and some key groups in terms of abundance were found only in burned sites. A notable example of a species found only in burned areas is the ant *Camponotus crassus* Mayr, 1862, a core species in interaction networks with other insects and plants (Belchior et al., 2023; Costa et al., 2016), which is highly abundant in open areas of *campo rupestre* (Castro et al., 2020; Perillo et al., 2021). Similarly, members of the *Ectatomma* genus followed the same trend, primarily foraging on the ground yet also exploiting the lower vegetation for foraging and nesting sites (Arruda et al., 2021). Perhaps, these species took advantage of a simplified habitat, potentially intensifying interactions between generalist ants and the lower vegetation, typically more frequent in burned sites (Swengel, 2001). Likewise, there is typically an over‐simplification of the litter stratum, which can benefit those groups that usually forage in open areas, such as *Ectatomma*, in detriment of those comprised of specific predators (e.g., *Pachycondyla*). Thus, our research provides crucial insights into the temporal dynamics of these insect groups in response to environmental changes and the influence of forest island connectivity through dispersal among habitats. Yet, we highlight the complex ecological responses and adaptations of insect communities, with great potential for shifts in species composition.

While we used vagility as a tenet explaining the differences between the different taxa in their responses to fire events, we must acknowledge that our approach has a few limitations. Critically, we did not perform direct measurements of the vagility of each specific species, and we acknowledge that there are a few exceptions within each studied taxon. For example, the diurnal large‐bodied dung beetle *Oxysternon conspicillatum* (Weber, 1801) can successfully fly over a few km when searching for food resources (Peck & Forsyth, 1982). Still, its group generally consists of species with low dispersal ability between vegetation patches and, thus, considered as having “low dispersal ability.” In addition, we advocate that future studies should also consider different aspects of the natural history of the organisms, including traits, interactions, and resource use specialization.

## 5 CONCLUSIONS

Ecological understanding of the anthropogenically induced effects on biota is essential if an attempt is to be made to mitigate the impact of human activity and conserve biodiversity. This is particularly true on islands, including forest islands, where communities of organisms are limited in their ability to cope with such impacts due to their small populations and the limited exchange of organisms between islands (Matthews, 2021). Indeed, focusing on forest islands, our findings underscore the vulnerability of specific insect taxa to fire incidence in forested systems (i.e., ants and dung beetles), while also revealing the resilience and adaptive capacity of others (i.e., bees, butterflies, and wasps). Potentially, under a scenario of global climate change, the loss of key species could lead to the disruption of interaction networks. This loss can then lead to a subsequent alteration of specific ecosystem processes and services that reverberate through countless trophic levels, impacting both the biotic and the abiotic components of the environment. Thus, bioindicator insects, as key players in numerous ecological processes, are instrumental in maintaining the overall functioning and stability of ecosystems (Tylianakis & Morris, 2017). By understanding and elucidating the intricate responses of distinct insect communities to global stressors, we can provide valuable insights for conservation efforts and environmental management strategies. We suggest that future research focus on long‐term, continuous monitoring (e.g., Record et al., 2021) of multitaxa groups to further investigate the underlying mechanisms behind the differential responses of insects to fire. These long‐term studies will also contribute to our understanding of the role of other anthropic disturbances in natural environments, taking into account factors such as habitat heterogeneity, microclimatic variations, and interactions between species. With this knowledge, policymakers, land managers, and conservationists can make informed decisions to protect and restore the biodiversity and ecological integrity of natural ecosystems.

## AUTHOR CONTRIBUTIONS

**Juliana Kuchenbecker:** Conceptualization (equal); investigation (equal); supervision (lead); writing – original draft (lead); writing – review and editing (lead). **Flávio Camarota:** Conceptualization (equal); supervision (equal); writing – original draft (equal); writing – review and editing (equal). **Pedro Giovâni da Silva:** Conceptualization (equal); formal analysis (equal); methodology (equal); writing – review and editing (equal). **Lucas Neves Perillo:** Methodology (equal); writing – review and editing (equal). **Marina do Vale Beirão:** Methodology (equal); writing – review and editing (equal). **Flávio Siqueira de Castro:** Methodology (equal); writing – review and editing (equal). **Geraldo Wilson Fernandes:** Funding acquisition (equal); writing – review and editing (equal). **Mário Marcos do Espírito‐Santo:** Writing – review and editing (equal). **Natália Correia Santos:** Writing – review and editing (supporting). **Iaciara Geórgia Silveira Cardoso:** Writing – review and editing (supporting). **Frederico de Siqueira Neves:** Conceptualization (lead); funding acquisition (equal); investigation (equal); methodology (equal); supervision (equal); writing – original draft (equal); writing – review and editing (equal).

## FUNDING INFORMATION

We are thankful to the *Conselho Nacional de Desenvolvimento Científico e Tecnológico* (CNPq) (PELD – 441515/2016‐9) for funding the long‐term ecological research “*PELD Campos Rupestres da Serra do Cipó*” and to the *Fundação de Amparo à Pesquisa do Estado de Minas Gerais* (Fapemig) for providing additional funding. JK thanks the INCT EECBio (*Ecologia, Evolução e Conservação da Biodiversidade*) and CNPq for a postdoctoral grant (380009/2023‐4). PGdS thanks the *Coordenação de Aperfeiçoamento de Pessoal de Nível Superior* (CAPES) for a postdoctoral grant (PNPD 88882.316025/2019‐01, Code 001). FC thanks CAPES for his postdoctoral grant (PRINT 88887.683127/2022‐00). FSN thanks CAPES for the fellowship grant (88887.888078/2023‐00). GWF thanks CNPq and Fapemig.

## CONFLICT OF INTEREST STATEMENT

The authors declare no competing interests.

## Supporting information


Data S1.

## Data Availability

The datasets generated during and/or analyzed during the current study are uploaded as a supporting material.
