# Supplementary material for: Differential response of fire on the community dynamics of five insect taxa in a tropical mountaintop forest archipelago
Source: Ecol Evol. 2023 Dec 6;13(12):e10806. doi: 10.1002/ece3.10806 (PMC10700047; doi:10.1002/ece3.10806)
Supplement: Supplementary file 1 — Data S1. [file ECE3-13-e10806-s001.docx]

**Supporting information**

**Fire increases temporal changes in species composition of less vagile insects in a mountaintop forest archipelago**

**Appendix S1.** Additional information on Material and Methods.

Material and Methods

Sampling design

**Bioindicator insects**

In this study, we used five insect taxa recognized as bioindicators: ants, bees, butterflies, dung beetles, and wasps. These insect groups utilize the resources within the study system differently and exhibit varying dispersal capacities and nesting strategies (da Silva et al., 2023a), which may contribute to their being distinctly affected by anthropic disturbances, such as fire.

1. **Ants**

Ants possess several characteristics that make them important bioindicators: high taxonomic diversity, abundance, and high number of interactions with other organisms (mostly other insects and plants), ease of sampling, and wide distribution across various habitats (Holldobler and Wilson, 1990; Lach et al., 2010). Furthermore, this insect group assumes great significance in maintaining ecosystem functions through the provision of vital ecosystem services such as litter decomposition, soil aeration, pollination, predation, and seed dispersal (Del-toro et al., 2012). Ants also serve as indicators of soil quality, making them exceptionally sensitive to anthropogenic impacts (Majer and Nichols, 1998; Underwood and Fisher, 2006).

1. **Bees**

Research has demonstrated that changes in bee behavior, population dynamics, and colony health provide early warning signs of environmental disturbances (Tsvetkov et al., 2017). Their ability to accumulate a wide range of environmental contaminants their role as reliable indicators of environmental pollution (Goulson et al., 2015). Ongoing investigations are exploring the potential application of bee monitoring as a tool for evaluate the overall condition and vitality of ecosystems and, thus, guiding conservation efforts (Quigley et al., 2019).

1. **Butterflies**

Butterflies draw attention due to their conspicuousness and ease of identification (Pollard and Yates,1993; Dennis, 2010; Dover et al., 2011). Many reports have demonstrated that butterfly communities' richness, abundance, and distribution are significantly influenced by different land uses (Nkongolo and Bapeamoni, 2018), forest disturbance (Lien and Yuan, 2003), and human activities (Kyerematen et al., 2018).

1. **Dung beetles**

Beetles, particularly dung beetles, have been extensively utilized in assessing habitat disturbance levels (Kaiser et al., 2009; Negro et al., 2010; Vasquez-Velez et al., 2010) and monitoring habitat management (Jacobs et al., 2010) and restoration (Babin-Fenske and Anand, 2010; Paoletti et al., 2010). They play an important role in ecosystem functioning by contributing to nutrient cycling, bioturbation, suppression of parasites and agricultural pests, trophic regulation, and secondary seed dispersal (Nichols et al., 2008). The fragmentation and isolation of forest patches significantly influence the distribution of this insect group in transformed landscapes (Nichols et al., 2007; Escobar et al., 2008, da Silva et al., 2023b), reiterating its sensitivity to minor environmental alterations in land use (Almeida et al. 2011).

1. **Wasps**

As predators and scavengers, and by ranking highly in food webs, wasps’ presence in a habitat indicates both the presence of an abundant and diverse community of prey arthropods (Gayubo et al., 2005), and subtle alterations in ecosystem dynamics (Gayubo et al., 2005; Henson et al., 2009). Some studies draw attention to regard social wasps as bioindicators highlighting changes not yet perceptible in plants and other animals (Dejean et al., 2011). Wasps, as well as the other groups of insects presented above, display heightened responsiveness to environmental stressors like habitat degradation (de Souza et al., 2010). Thus, studying wasp populations provides valuable insights into the impacts of human activities on forest ecosystems and enables the early and ongoing detection of environmental disturbances (Restello & Penteado-Dias, 2006; de Souza et al., 2010).

References

Almeida S, Louzada J, Sperber C, Barlow J (2011) Subtle land-use change and tropical biodiversity: dung beetle communities in cerrado grasslands and exotic pastures. Biotropica 43:704–710

Andersen AN (1997) Using ants as bioindicators: multi-scale issues in ant community ecology. Conser Ecol 1(1):8

Antonio, J. R., M. Gutierrez, and C. Porrini. 2013. Biomonitoring of bees as bioindicators. Bee World 90 (3):61–3. doi: 10.1080/0005772X.2013.11417545.

Babin-Fenske J, Anand M (2010) Terrestrial insect communities and the restoration of an industrially perturbed landscape: assessing success and surrogacy. Restor Ecol 18:73–84

Barganska, Z., M. Slebioda, and J. Namiesnik. 2016. Honey bees and their products - Bioindicators of environmental contamination. Critical Reviews in Environmental Science and Technology 46 (3):235–28. doi: 10.1080/10643389.2015.1078220.

Bichara, C.C., G.M.M. Santos, J.J. Resende, D.J. Cruz, N. Gobbi & V.L.L. Machado 2009. Foraging behavior of the swarm-founding wasp, Polybia (Trichothorax) sericea (Hymenoptera: Vespidae): prey capture and load capacity. Sociobiology 53(1):61-69.

Bisevac L, Majer JD (1999) Comparative study of ant communities of rehabilitated mineral sand mines and heathland, Western Aus- tralia. Restorat Ecol 7:117–126

Chowdhury, S., Dubey, V. K., Choudhury, S., Das, A., Jeengar, D., Sujatha, B., ... & Kumar, V. (2023). Insects as bioindicator: A hidden gem for environmental monitoring. Frontiers in Environmental Science, 11, 273.

da Silva, P.G., Nunes, C.A., Ferreira, L.F., Braga, R.F., Beiroz, W., Perillo, L.N., Solar, R.R.C., Neves, F.S., 2019. Patch and landscape effects on forest-dependent dung beetles are masked by matrix-tolerant dung beetles in a mountaintop rainforest archipelago. Sci. Total Environ. 651, 1321-1331. <https://doi.org/10.1016/j.scitotenv.2018.09.195>

da Silva et al., 2023a

da Silva, P.G., Salomão, R.P., González-Tokman, D., Neves, F.S. & Favila, M.E. (2023b) Temporal changes of taxonomic and functional diversity in dung beetles inhabiting forest fragments and pastures in Los Tuxtlas Biosphere Reserve, Mexico. Revista Mexicana de Biodiversidad, 94, e945059.

de Souza, M. M., Louzada, J., Serrão, J. E. & Zanuncio, J. C. (2010). Social wasps (Hymenoptera: Vespidae) as indicators of conservation degree of riparian forests in Southeast Brazil. Sociobiology 56, 387– 396.

Dejean, A., Cereghino, R., Carpenter, J. M., Corbara, B., Herault, B., Rossi, V., ... & Bonal, D. (2011). Climate change impact on Neotropical social wasps. PLoS One, 6(11), e27004.

Del Toro, I., Ribbons, R. R., & Pelini, S. L. (2012). The little things that run the world revisited: a review of ant-mediated ecosystem services and disservices (Hymenoptera: Formicidae). Myrmecological News, 17(0), 133-46.

Dennis RLH (2010) A resource-based habitat view for conservation. Butterflies in the British landscape. Wiley-Blackwell, London

Dover J, Warren M, Shreeve T (eds) (2011) Lepidoptera conservation in a changing world. Springer, Dordrecht

Escobar F, Halffter G, Sol ́ıs A ́, Halffter V, Navarrete D (2008) Temporal shifts in dung beetle community structure within a protected area of tropical wet forest: a 35-year study and its implications for long-term conservation. J Appl Ecol 45: 1584–1592

GardnerTA,Herna ́ndezMIM,BarlowJ,PeresCA(2007)Under- standing the biodiversity consequences of habitat change: the value of secondary and plantation forests for neotropical dung beetles. J Appl Ecol 45:883–893

Gayubo, S. F., González, J. A., Asís, J. D. & Tormos, J. (2005). Conservation of European environments: the Spheciformes wasps as biodiversity indicators (Hymenoptera: Apoidea: Ampulicidae, Sphecidae and Crabronidae). Journal of Natural History 39, 2705– 2714.

Gerlach, J., Samways, M., & Pryke, J. (2013). Terrestrial invertebrates as bioindicators: an overview of available taxonomic groups. Journal of insect conservation, 17, 831-850.

Goulson, D., Nicholls, E., Botías, C., & Rotheray, E. L. (2015). Bee declines driven by combined stress from parasites, pesticides, and lack of flowers. *Science*. https://doi.org/1255957

Henson, K. S. E., Craze, P. G. & Memmott, J. (2009). The restoration of parasites, parasitoids, and pathogens to heathland communities. Ecology 90, 1840– 1851.

Hölldobler, B., & Wilson, E. O. (1990). The ants. Harvard University Press.

Jacobs CT, Scholtz CH, Escobar F, Davis ALV (2010) How might intensification of farming influence dung beetle diversity (Cole- optera: Scarabaeidae) in Maputo Special Reserve (Mozam- bique)? J Insect Conserv 14:389–399

Kaiser W, Avenant NL, Haddad CR (2009) Assessing the ecological integrity of a grassland ecosystem: the applicability and rapidity of the SAGraSS method. Afr J Ecol 47:308–317

Kyerematen, R., Adu-Acheampong, S., Acquah-Lamptey, D., Sigismund, R. A., Owusu, E. H., & Mantey, J. (2018). Butterfly Diversity: An Indicator For Environmental Health Withintarkwa Gold Mine, Ghana.

Lach, L., Parr, C., & Abbott, K. (Eds.). (2010). Ant ecology. Oxford university press.McGeoch, M. A. (2007). Insects and bioindication: theory and progress. Insect conservation biology, 144-174.

Lien V, Yuan D (2003).The differences of butterfly (Lepidoptera, Papilionoidea) communities in habitats with various degrees of disturbance and altitudes in tropical forests of Vietnam. Biodivers. Conserv. 12:1099-1111

Majer JD, de Kock AE (1992) Ant recolonization of sand mines near Richards Bay, South Africa: an evaluation of progress with rehabilitation. S Afr J Sci 88:31–36

Majer, J. D., and O. G. Nichols. 1998. Long-term recolonization patterns of ants in Western Australian rehabilitated bauxite mines with reference to their use as indicators of restoration success. Journal of Applied Ecology 35 (1):161–82. doi: 10.1046/j.1365- 2664.1998.00286.x.

Majer, J. D. 1983. Ants: Bio-indicators of mine site rehabilitation, land-use, and land con- servation. Environmental Management 7 (4):375–83. doi: 10.1007/BF01866920.

Majer JD (1992) Ant recolonization of rehabilitated bauxite mines of Pocos de Caldas, Brazil. J Trop Ecol 8:97–108

McGeoch MA (2007) Insects and bioindication: theory and progress. In: Stewart AJA, New TR, Lewis OT (eds) Insect conservation biology. Proceedings of the royal entomological society’s 23rd symposium. CAB International, Wallingford, pp 144–174

McGeoch MA, Sithole H, Samways MJ, Simaika JP, Pryke JS, Picker M, Uys C, Armstrong AJ, Dippenaar-Schoeman AS, Engelbrecht IA, Braschler B, Hamer M (2011) Conservation and monitoring of invertebrates in terrestrial protected areas. Koedoe 53:1–13

Negro M, Isaia M, Palestrini C, Schoenhofer A, Rolando A (2010) The impact of high-altitude ski pistes on ground-dwelling arthropods in the Alps. Biodiv Conserv 19(7):1853–1870

Nichols E, Larsen T, Spector S, Davis AL, Escobar F, Favila M, Vuline K (2007) Global dung beetle response to tropical forest modification and fragmentation: a quantitative literature review and meta-analysis. Biol Conserv 137:1–19

Nichols, E., Spector, S., Louzada, J., Larsen, T., Amezquita, S., Favila, M. E., & Network, T. S. R. (2008). Ecological functions and ecosystem services provided by Scarabaeinae dung beetles. Biological conservation, 141(6), 1461-1474.

Nkongolo, N. V., & Bapeamoni, F. (2018). The effect of land use type on butterfly diversity at Masako Forest Reserve, Kisangani, Democratic Republic of Congo. International Journal of Biodiversity and Conservation, 10(3), 131-144.

Paoletti MG, D’Inca A, Tonin E, Tonon S, Migliorini C, Petruzzelli G, Pezzarossa B, Gomiero T, Sommaggio D (2010) Soil invertebrates as bio-indicators in a natural area converted from agricultural use: the case study of Vallevecchia-Lugugnana in north-eastern Italy. J Sust Agric 34:38–56

Paoletti, M. G. (2012). Invertebrate biodiversity as bioindicators of sustainable landscapes: Practical use of invertebrates to assess sustainable land use. Elsevier.

Parikh, G., Rawtani, D., & Khatri, N. (2021). Insects as an indicator for environmental pollution. Environmental Claims Journal, 33(2), 161-181.

Pollard E, Yates TJ (1993) Monitoring butterflies for ecology and conservation. Joint Nature Conservation Committee Monks Wood, UK

Prezoto, F., H.H. Santos-Prezoto, V.L.L. Machado & J.C. Zanuncio 2006. Prey captured and usedinPolistesversicolor(Olivier)(Hymenoptera:Vespidae)nourishment.Neotropical Entomology 35(5):707-709.

Quigley, T. P., Amdam, G. V., & Harwood, G. H. (2019). Honey bees as bioindicators of changing global agricultural landscapes. Current opinion in insect science, 35, 132-137.

Restello, R.S. & A.M. Penteado-Dias 2006. Diversidade dos Braconidae (Hymenoptera) da unidadedeconservaçãodeTeixeiraMarcelinoRamos,RS,comênfasenosMicrogastrinae. Revista Brasileira de Entomologia 50(1):80-84.

Samways MJ, McGeoch MA, New TR (2010a) Insect conservation: a handbook of approaches and methods. Oxford University Press,Oxford

Tsvetkov, N., Samson-Robert, O., Sood, K., Patel, H. S., Malena, D. A., Gajiwala, P. H., Maciukiewicz, P., Fournier, V., & Zayed, A. (2017). Chronic exposure to neonicotinoids reduces honey bee health near corn crops. *Science*. <https://doi.org/aam7470>

Underwood, E. C., and B. L. Fisher 2006. The role of ants in conservation monitoring: If, when, and how. Biological Conservation 132:166–182.

Vasquez-Velez LM, Bermudez C, Chacon P, Lozano-Zambrano FH (2010) Analysis of the richness of Staphylinidae (Coleoptera) on different scales of a sub-Andean rural landscape in Colombia. Biodiv Conserv 19:1917–1931

**Table S1.** An overview of each sampled forest island, where PERIM refers to the Perimeter; and NFI is the distance from the nearest forest island measured from the center point of each island.

| Forest Island | Elevation (m) | Landscape Metrics year 2020 | | | Fire records | Fire frequency |
| --- | --- | --- | --- | --- | --- | --- |
|  |  | AREA (m2) | PERIM (m) | NFI (m) |  |  |
| FI 1 | 1239 | 1,0972 | 522 | 379 | absent | 0 |
| FI 2 | 1262 | 3,8453 | 1148 | 331 | absent | 0 |
| FI 3 | 1234 | 1,5334 | 978 | 331 | absent | 0 |
| FI 4 | 1269 | 16,5197 | 2668 | 1270 | absent | 0 |
| FI 5 | 1309 | 2,4541 | 1006 | 1530 | present | 2 |
| FI 6 | 1317 | 9,1724 | 2300 | 575 | absent | 0 |
| FI 7 | 1331 | 5,9051 | 1436 | 200 | absent | 0 |
| FI 8 | 1271 | 6,3416 | 2104 | 1416 | present | 1 |
| FI 9 | 1230 | 37,6007 | 4182 | 1864 | absent | 0 |
| FI 10 | 1324 | 6,564 | 1340 | 1255 | present | 1 |
| FI 11 | 1273 | 0,8314 | 604 | 264 | present | 1 |
| FI 12 | 1267 | 0,8527 | 508 | 264 | present | 1 |
| FI 13 | 1290 | 1,3759 | 970 | 200 | present | 1 |
| FI 14 | 1235 | 9,0769 | 2156 | 1590 | absent | 0 |

**Table S2.** Species of ants, bees, butterflies, dung beetles, and wasps sampled in the forest archipelago in the Espinhaço Mountain Range, Brazil. T = total.

| **Group** | **Species** | **Forest islands** | | | | | | | | | | | | | |  | **Years** | | | |
| --- | --- | --- | --- | --- | --- | --- | --- | --- | --- | --- | --- | --- | --- | --- | --- | --- | --- | --- | --- | --- |
|  |  | **1** | **2** | **3** | **4** | **5** | **6** | **7** | **8** | **9** | **10** | **11** | **12** | **13** | **14** |  | **2014** | **2015** | **2018** | **2020** |
| Ant | *Acromyrmex coronata* |  |  | 1 |  |  |  |  |  |  |  |  |  |  |  |  |  |  | 1 |  |
|  | *Acromyrmex subterraneus* | 1 | 1 | 1 | 1 | 1 | 1 | 1 | 1 | 1 | 1 | 1 | 1 | 1 | 1 |  | 1 | 1 | 1 | 1 |
|  | *Anochetus altisquamis* | 1 |  |  |  |  |  |  | 1 | 1 |  |  |  |  |  |  | 1 | 1 |  | 1 |
|  | *Anochetus inermis* |  |  |  |  |  |  |  | 1 | 1 |  |  |  |  |  |  | 1 | 1 |  |  |
|  | *Apterostigma pilosum* | 1 | 1 | 1 | 1 |  | 1 | 1 |  |  | 1 | 1 | 1 |  |  |  | 1 | 1 | 1 | 1 |
|  | *Basiceros disciger* |  | 1 |  | 1 |  |  |  |  |  | 1 |  |  |  |  |  | 1 | 1 |  |  |
|  | *Brachymyrmex* sp.1 | 1 | 1 |  | 1 |  | 1 | 1 | 1 | 1 | 1 | 1 | 1 | 1 | 1 |  | 1 | 1 | 1 | 1 |
|  | *Brachymyrmex* sp.3 |  | 1 | 1 |  |  |  |  |  |  |  |  | 1 | 1 |  |  |  | 1 |  | 1 |
|  | *Brachymyrmex* sp.5 |  |  |  |  |  |  |  |  | 1 |  |  |  |  |  |  | 1 | 1 |  |  |
|  | *Brachymyrmex* sp.6 |  |  |  |  |  | 1 |  |  | 1 |  |  |  |  |  |  |  | 1 | 1 | 1 |
|  | *Camponotus (Hypercolobopsis)* sp.1 |  |  |  |  |  |  |  | 1 |  |  |  |  |  |  |  |  |  |  | 1 |
|  | *Camponotus* *arboreus* |  |  |  |  |  |  |  | 1 |  |  |  |  |  |  |  |  |  |  | 1 |
|  | *Camponotus* cf. *sexgutattus* |  |  | 1 |  |  | 1 | 1 |  |  |  | 1 |  |  | 1 |  | 1 | 1 | 1 | 1 |
|  | *Camponotus* cf. *blandus* |  |  | 1 |  | 1 |  |  | 1 |  |  |  |  |  |  |  | 1 | 1 | 1 | 1 |
|  | *Camponotus* *cingulatus* |  | 1 | 1 |  |  |  |  | 1 |  |  |  | 1 |  |  |  | 1 |  | 1 |  |
|  | *Camponotus crassus* |  |  |  |  | 1 | 1 |  |  |  | 1 | 1 |  | 1 |  |  |  | 1 | 1 |  |
|  | *Camponotus fastigatus* |  |  |  | 1 | 1 |  |  | 1 |  |  |  |  | 1 |  |  |  |  | 1 | 1 |
|  | *Camponotus lespesii* | 1 | 1 | 1 | 1 | 1 | 1 | 1 | 1 | 1 | 1 | 1 | 1 | 1 | 1 |  | 1 | 1 | 1 | 1 |
|  | *Camponotus melanoticus* |  | 1 | 1 | 1 | 1 | 1 | 1 | 1 | 1 |  | 1 | 1 | 1 | 1 |  | 1 | 1 | 1 |  |
|  | *Camponotus novogranadensis* | 1 | 1 |  | 1 |  | 1 |  | 1 |  |  | 1 |  |  | 1 |  | 1 | 1 | 1 |  |
|  | *Camponotus rufipes* | 1 | 1 | 1 | 1 | 1 | 1 | 1 | 1 | 1 | 1 | 1 | 1 | 1 | 1 |  | 1 | 1 | 1 | 1 |
|  | *Camponotus* sp.4 | 1 | 1 |  | 1 |  | 1 | 1 |  |  | 1 | 1 |  | 1 |  |  | 1 | 1 | 1 | 1 |
|  | *Camponotus* sp.25 |  |  |  |  | 1 |  |  |  |  |  | 1 |  |  |  |  |  | 1 |  |  |
|  | *Camponotus* sp.48 |  | 1 |  |  |  | 1 |  |  |  | 1 |  |  |  |  |  |  |  | 1 | 1 |
|  | *Camponotus tripartitus* | 1 | 1 | 1 |  | 1 | 1 |  |  | 1 |  |  |  | 1 |  |  | 1 | 1 | 1 |  |
|  | *Carebara* sp.3 |  |  |  | 1 |  |  |  |  |  | 1 |  |  |  |  |  |  | 1 |  |  |
|  | *Carebara* sp.4 |  |  |  |  |  |  |  | 1 |  |  |  |  |  |  |  |  | 1 |  |  |
|  | *Cephalotes pusillus* |  |  |  |  |  |  |  |  | 1 |  |  |  |  |  |  |  |  | 1 |  |
|  | *Crematogaster brasiliensis* |  |  |  | 1 |  |  |  |  | 1 |  |  | 1 |  |  |  | 1 | 1 | 1 | 1 |
|  | *Crematogaster erecta* |  |  | 1 |  |  |  | 1 |  |  |  |  |  |  |  |  |  | 1 | 1 |  |
|  | *Crematogaster* sp.7 |  |  |  |  |  |  |  |  |  |  |  | 1 |  |  |  | 1 |  |  |  |
|  | *Crematogaster* sp.8 |  |  |  |  |  |  |  | 1 |  |  |  |  |  |  |  |  | 1 |  |  |
|  | *Crematogaster torosa* |  |  |  |  | 1 |  |  |  |  |  |  | 1 | 1 |  |  | 1 | 1 | 1 |  |
|  | *Cyphomyrmex* cf. *dixus* | 1 | 1 |  | 1 |  | 1 | 1 |  |  | 1 | 1 |  |  | 1 |  | 1 | 1 |  |  |
|  | *Cyphomyrmex hamulatus* | 1 |  | 1 |  |  | 1 |  |  | 1 | 1 | 1 | 1 |  |  |  | 1 | 1 |  |  |
|  | *Cyphomyrmex* *minutus* |  |  |  | 1 |  |  |  | 1 | 1 | 1 | 1 | 1 | 1 |  |  | 1 | 1 | 1 | 1 |
|  | *Cyphomyrmex* *rimosus* | 1 |  |  | 1 |  | 1 |  |  | 1 | 1 | 1 | 1 | 1 | 1 |  | 1 | 1 | 1 | 1 |
|  | *Dorymyrmex brunneus* |  | 1 |  |  |  |  |  |  |  |  |  |  |  |  |  |  | 1 |  |  |
|  | *Eciton harmatum* |  |  |  |  |  |  |  |  |  |  |  |  | 1 |  |  |  |  | 1 |  |
|  | *Ectatomma edentatum* |  |  |  |  |  |  |  | 1 |  |  | 1 |  |  |  |  |  |  | 1 | 1 |
|  | *Ectatomma permagnum* |  |  |  |  |  |  |  | 1 |  |  |  |  |  |  |  |  |  | 1 |  |
|  | *Fulakora* sp.1 |  |  |  |  |  |  |  |  |  |  |  |  | 1 |  |  | 1 |  |  |  |
|  | *Gnamptogenys* sp.5 |  |  |  |  |  | 1 |  |  |  | 1 |  |  |  |  |  |  | 1 |  |  |
|  | *Gnamptogenys sulcata* |  |  |  |  |  |  |  |  |  |  | 1 |  |  | 1 |  | 1 |  | 1 |  |
|  | *Heteroponera dentinodus* |  | 1 |  | 1 |  |  |  |  | 1 |  |  |  |  |  |  | 1 | 1 |  | 1 |
|  | *Holcoponera striatula* | 1 | 1 | 1 | 1 | 1 | 1 | 1 | 1 | 1 | 1 | 1 | 1 | 1 | 1 |  | 1 | 1 | 1 | 1 |
|  | *Hylomyrma balzani* | 1 | 1 | 1 | 1 |  |  | 1 | 1 | 1 |  | 1 | 1 | 1 | 1 |  | 1 | 1 | 1 |  |
|  | *Holcoponera mina* |  |  |  |  |  |  |  |  |  | 1 | 1 |  |  |  |  |  |  | 1 | 1 |
|  | *Hylomyrma reitteri* |  |  |  | 1 |  |  | 1 |  | 1 |  | 1 |  |  | 1 |  | 1 | 1 |  |  |
|  | *Hypoponera* sp.2 | 1 | 1 | 1 | 1 | 1 | 1 | 1 | 1 | 1 |  | 1 | 1 | 1 |  |  | 1 | 1 | 1 |  |
|  | *Hypoponera* sp.3 | 1 | 1 | 1 | 1 |  |  |  |  |  | 1 | 1 | 1 | 1 |  |  | 1 | 1 | 1 | 1 |
|  | *Hypoponera* sp.4 |  |  |  |  | 1 |  |  | 1 | 1 |  |  |  | 1 |  |  |  | 1 | 1 | 1 |
|  | *Labidus* *praedator* |  |  |  |  |  | 1 |  |  |  |  |  |  |  | 1 |  | 1 | 1 |  |  |
|  | *Leptogenys* sp.1 |  |  |  |  |  |  |  |  |  |  |  | 1 |  |  |  |  | 1 |  |  |
|  | *Linepithema aztecoides* | 1 | 1 | 1 |  | 1 |  |  | 1 |  |  |  |  | 1 |  |  |  | 1 |  | 1 |
|  | *Linepithema cerradense* | 1 |  |  | 1 |  |  |  | 1 | 1 |  | 1 |  |  |  |  |  |  | 1 | 1 |
|  | *Linepithema iniquum* | 1 | 1 |  |  |  |  |  |  |  | 1 | 1 | 1 | 1 | 1 |  | 1 | 1 | 1 |  |
|  | *Linepithema leucomelas* | 1 |  | 1 |  |  |  | 1 |  |  |  |  |  | 1 | 1 |  | 1 | 1 | 1 | 1 |
|  | *Linepithema micans* | 1 | 1 | 1 |  | 1 |  |  | 1 | 1 | 1 |  | 1 |  |  |  | 1 | 1 | 1 | 1 |
|  | *Linepithema neotropicum* |  | 1 |  |  | 1 |  |  | 1 |  | 1 |  | 1 |  | 1 |  |  | 1 |  |  |
|  | *Linepithema* *pulex* | 1 |  | 1 | 1 | 1 |  | 1 | 1 | 1 |  |  |  | 1 | 1 |  | 1 | 1 | 1 | 1 |
|  | *Mycetomoellerius* sp.7 | 1 |  |  |  |  | 1 |  |  |  | 1 | 1 |  | 1 | 1 |  | 1 | 1 | 1 | 1 |
|  | *Mycetophylax* *strigatus* sp.5 |  | 1 |  |  |  |  |  |  |  |  |  |  |  |  |  |  | 1 |  |  |
|  | *Myrmelachista catharinae* | 1 |  |  |  | 1 | 1 | 1 |  | 1 | 1 |  | 1 | 1 |  |  | 1 | 1 | 1 | 1 |
|  | *Myrmelachista nodigera* |  |  |  |  |  |  |  |  |  |  |  |  | 1 |  |  |  |  | 1 |  |
|  | *Myrmelachista* sp.6 |  |  |  |  |  | 1 |  |  |  |  |  |  |  |  |  |  | 1 |  |  |
|  | *Neivamyrmex* sp.2 |  |  | 1 |  | 1 |  |  |  |  |  |  |  |  |  |  |  | 1 |  |  |
|  | *Neivamyrmex* sp.5 |  |  |  |  |  |  |  |  |  |  | 1 |  |  |  |  | 1 |  |  |  |
|  | *Neocerapachys splendens* |  |  |  |  |  |  |  |  |  | 1 |  |  |  |  |  |  | 1 |  |  |
|  | *Neoponera crenata* | 1 |  | 1 |  | 1 |  |  | 1 |  |  |  |  |  |  |  |  | 1 | 1 | 1 |
|  | *Neoponera laevigatta* |  |  |  |  | 1 |  |  | 1 | 1 |  |  |  |  |  |  |  |  | 1 | 1 |
|  | *Neoponera verenae* |  |  |  |  |  |  |  | 1 |  |  |  |  |  |  |  |  |  |  | 1 |
|  | *Nylanderia* sp.1 | 1 | 1 | 1 | 1 | 1 |  |  | 1 | 1 | 1 | 1 | 1 | 1 | 1 |  | 1 | 1 | 1 | 1 |
|  | *Octostruma balzani* |  |  |  |  |  | 1 |  |  |  |  |  |  |  |  |  |  |  | 1 |  |
|  | *Octostruma rugifera* | 1 |  |  |  |  | 1 |  |  |  |  |  |  |  |  |  | 1 |  |  |  |
|  | *Octostruma* sp.1 |  |  | 1 |  |  |  |  |  |  |  |  |  |  |  |  |  | 1 |  |  |
|  | *Odontomachus* cf. *haematodus* |  |  |  |  |  |  |  |  |  |  |  |  | 1 |  |  | 1 | 1 |  | 1 |
|  | *Oxyepoecus* sp.3 |  |  |  |  | 1 |  | 1 |  |  |  |  |  |  |  |  |  |  | 1 | 1 |
|  | *Oxyepoecus* sp.4 |  |  |  |  |  |  |  |  |  |  |  | 1 | 1 |  |  |  |  | 1 |  |
|  | *Oxyepoecus* sp.5 |  |  |  |  |  | 1 |  |  |  |  |  |  | 1 |  |  | 1 | 1 |  |  |
|  | *Pachycondyla harpax* |  | 1 |  | 1 | 1 |  |  | 1 | 1 | 1 | 1 | 1 | 1 | 1 |  | 1 | 1 | 1 | 1 |
|  | *Pachycondyla striata* | 1 | 1 | 1 | 1 | 1 | 1 | 1 | 1 | 1 | 1 | 1 | 1 | 1 | 1 |  | 1 | 1 | 1 | 1 |
|  | *Pheidole jelskii* | 1 | 1 | 1 | 1 | 1 | 1 | 1 | 1 | 1 | 1 | 1 | 1 | 1 | 1 |  | 1 | 1 | 1 | 1 |
|  | *Pheidole oxyops* | 1 | 1 | 1 | 1 | 1 | 1 | 1 | 1 | 1 | 1 | 1 | 1 | 1 | 1 |  | 1 | 1 | 1 | 1 |
|  | *Pheidole radoszkowskii* |  |  | 1 |  | 1 | 1 |  | 1 | 1 | 1 |  | 1 | 1 | 1 |  | 1 | 1 | 1 | 1 |
|  | *Pheidole* sp.2 |  | 1 |  | 1 | 1 | 1 | 1 | 1 | 1 | 1 | 1 | 1 | 1 | 1 |  | 1 | 1 | 1 | 1 |
|  | *Pheidole* sp.7 |  |  |  |  |  |  | 1 |  |  |  |  |  |  |  |  |  |  | 1 |  |
|  | *Pheidole* sp.8 | 1 | 1 | 1 | 1 | 1 | 1 | 1 | 1 | 1 | 1 | 1 | 1 | 1 | 1 |  | 1 | 1 | 1 | 1 |
|  | *Pheidole* sp.13 |  | 1 | 1 | 1 | 1 | 1 | 1 | 1 | 1 | 1 | 1 | 1 | 1 | 1 |  | 1 | 1 | 1 | 1 |
|  | *Pheidole* sp.48 |  | 1 |  |  |  |  |  |  | 1 | 1 |  |  |  |  |  | 1 | 1 |  |  |
|  | *Pheidole* sp.49 |  |  |  |  |  |  |  |  |  |  |  |  |  | 1 |  | 1 |  |  |  |
|  | *Pheidole* sp.50 |  |  |  |  | 1 |  |  | 1 | 1 |  |  |  |  |  |  | 1 | 1 |  | 1 |
|  | *Pheidole* sp.54 |  |  |  | 1 |  | 1 |  | 1 | 1 | 1 |  |  |  |  |  | 1 | 1 | 1 | 1 |
|  | *Pheidole subarmata* |  | 1 | 1 |  | 1 |  |  |  |  |  | 1 |  |  |  |  | 1 | 1 | 1 |  |
|  | *Pogonomyrmex naegelii* |  |  |  |  | 1 |  |  |  |  |  |  |  |  | 1 |  |  | 1 | 1 |  |
|  | *Procryptocerus montanus* |  | 1 |  |  |  |  |  |  |  |  | 1 | 1 |  | 1 |  |  | 1 | 1 | 1 |
|  | *Procryptocerus* sp.1 |  |  |  |  |  |  |  |  |  |  |  |  |  | 1 |  |  | 1 |  |  |
|  | *Pseudomyrmex gracilis* |  |  |  |  |  |  |  |  | 1 | 1 |  |  |  |  |  | 1 |  |  | 1 |
|  | *Pseudomyrmex* sp4 |  |  |  | 1 | 1 |  | 1 | 1 | 1 |  |  |  |  | 1 |  | 1 | 1 | 1 | 1 |
|  | *Pseudomyrmex* sp.7 |  | 1 | 1 |  |  |  | 1 | 1 | 1 |  | 1 |  |  |  |  | 1 | 1 | 1 | 1 |
|  | *Pseudomyrmex* sp.8 |  |  |  |  |  |  |  |  | 1 |  |  |  |  |  |  | 1 |  |  |  |
|  | *Rogeria* sp.2 |  |  | 1 |  |  |  |  |  |  |  | 1 |  |  |  |  | 1 |  |  |  |
|  | *Solenopsis* sp.1 | 1 | 1 | 1 | 1 | 1 | 1 | 1 | 1 | 1 | 1 | 1 | 1 | 1 | 1 |  | 1 | 1 | 1 | 1 |
|  | *Solenopsis* sp.2 |  |  |  |  | 1 |  |  |  |  |  |  | 1 | 1 |  |  | 1 |  |  |  |
|  | *Solenopsis* sp.7 | 1 | 1 |  | 1 | 1 | 1 | 1 |  | 1 | 1 | 1 | 1 | 1 | 1 |  | 1 | 1 | 1 | 1 |
|  | *Solenopsis* sp.8 |  |  |  |  | 1 |  | 1 |  |  |  |  |  |  | 1 |  | 1 | 1 | 1 |  |
|  | *Solenopsis* sp.9 |  |  |  |  |  |  | 1 |  |  |  |  |  |  |  |  |  |  |  | 1 |
|  | *Sphinctomyrmex* cf. *stali* |  |  |  |  |  |  |  |  |  | 1 |  |  |  |  |  | 1 |  |  |  |
|  | *Strumigenys* *denticulata* |  |  |  | 1 |  |  |  |  |  |  |  |  |  |  |  | 1 |  |  |  |
|  | *Strumigenys* gr. *hadrodens* |  |  |  | 1 |  |  |  |  |  |  |  |  |  |  |  |  |  | 1 |  |
|  | *Strumigenys* sp.4 |  |  |  |  |  |  |  |  |  |  |  |  |  | 1 |  |  | 1 |  |  |
|  | *Tapinoma atriceps* |  | 1 |  |  |  |  | 1 |  |  |  |  |  |  |  |  |  | 1 |  |  |
|  | *Typhlomyrmex* sp.1 |  |  |  |  |  |  |  |  |  |  |  |  | 1 |  |  |  | 1 |  |  |
|  | *Wasmannia lutzi* |  | 1 | 1 | 1 |  | 1 | 1 | 1 | 1 | 1 | 1 | 1 | 1 | 1 |  | 1 | 1 | 1 | 1 |
| Bee | Apinae sp.01 |  |  |  |  |  |  |  |  |  |  |  |  |  | 1 |  | 1 |  |  |  |
|  | Apinae sp.02 |  |  |  |  | 1 |  |  |  |  |  |  |  |  |  |  |  | 1 |  |  |
|  | *Apis mellifera* | 1 | 1 |  | 1 |  |  |  | 1 | 1 |  |  | 1 | 1 |  |  | 1 | 1 | 1 | 1 |
|  | Augochlorini sp.01 |  |  |  |  | 1 |  |  |  |  |  |  |  |  |  |  |  | 1 |  |  |
|  | Augochlorini sp.02 |  |  |  |  |  |  |  |  |  |  |  |  |  | 1 |  |  | 1 |  |  |
|  | Augochlorini sp.04 |  |  |  |  |  |  | 1 |  | 1 |  |  |  |  |  |  |  | 1 | 1 |  |
|  | Augochlorini sp.05 |  |  |  |  |  |  |  |  |  |  |  |  |  | 1 |  | 1 |  |  |  |
|  | Augochlorini sp.06 |  |  |  |  | 1 |  |  |  |  |  |  |  |  |  |  | 1 |  |  |  |
|  | *Augochloropsis hebescens* |  |  |  |  |  |  |  |  |  |  |  |  |  | 1 |  | 1 |  |  |  |
|  | *Augochloropsis* sp.01 |  |  |  |  |  |  |  |  |  |  |  |  | 1 |  |  |  |  |  | 1 |
|  | *Augochloropsis* sp.02 |  |  |  |  |  |  |  |  |  |  |  |  | 1 |  |  |  |  |  | 1 |
|  | *Centris* sp.01 |  |  |  |  |  |  |  | 1 |  |  |  |  |  |  |  |  |  | 1 |  |
|  | *Ceratina (Ceratinula)* sp.01 |  |  | 1 |  |  | 1 |  |  |  |  |  | 1 |  | 1 |  | 1 | 1 |  |  |
|  | *Ceratina (Ceratinula)* sp.02 |  |  |  |  | 1 |  |  |  |  |  |  |  |  |  |  | 1 |  |  |  |
|  | *Ceratina (Ceratinula)* sp.03 |  |  | 1 |  |  |  |  |  |  |  |  |  |  | 1 |  | 1 | 1 |  |  |
|  | *Ceratina (Ceratinula)* sp.05 |  |  |  |  | 1 |  |  |  |  |  |  |  |  |  |  |  | 1 |  |  |
|  | *Ceratina (Ceratinula)* sp.06 |  |  |  |  |  |  |  |  |  |  |  |  |  | 1 |  | 1 |  |  |  |
|  | *Ceratina (Ceratinula)* sp.07 |  |  |  | 1 |  |  |  | 1 |  | 1 |  | 1 |  | 1 |  | 1 |  | 1 |  |
|  | *Ceratina (Ceratinula)* sp.08 |  |  |  |  | 1 |  |  |  |  |  |  |  |  |  |  |  | 1 |  |  |
|  | *Ceratina (Crewella)* sp.01 |  |  |  |  | 1 |  |  |  |  |  |  |  |  |  |  |  | 1 |  |  |
|  | Halictini sp.01 |  |  |  |  |  |  |  |  |  |  |  | 1 |  |  |  |  | 1 |  |  |
|  | *Hylaeus* sp. |  | 1 |  |  |  |  |  |  |  | 1 |  |  |  |  |  | 1 | 1 |  |  |
|  | *Lasioglossum (Dialictus)* sp.01 |  |  | 1 |  |  |  |  |  |  |  |  |  | 1 |  |  | 1 | 1 |  |  |
|  | *Lasioglossum (Dialictus)* sp.03 |  |  | 1 |  |  |  |  |  |  |  |  |  |  |  |  |  | 1 |  |  |
|  | *Lasioglossum (Dialictus)* sp.04 |  |  |  |  | 1 |  |  |  |  |  | 1 | 1 | 1 |  |  |  | 1 |  |  |
|  | *Lasioglossum (Dialictus)* sp.08 |  |  |  |  |  |  |  |  |  |  |  |  |  | 1 |  |  | 1 |  |  |
|  | *Melipona (Melipona) quadrifasciata* | 1 | 1 | 1 |  | 1 | 1 |  |  | 1 |  | 1 | 1 | 1 | 1 |  | 1 | 1 | 1 | 1 |
|  | *Melitoma torquata* |  |  |  |  | 1 |  |  |  |  |  |  |  |  |  |  | 1 |  |  |  |
|  | *Neocorynura aenigma* |  |  |  |  |  |  |  |  |  |  |  |  |  | 1 |  | 1 | 1 |  |  |
|  | *Paratrigona lineata* |  |  |  |  |  |  |  |  |  |  |  |  |  | 1 |  | 1 |  |  |  |
|  | *Partamona ailyae* |  |  |  | 1 |  |  |  |  |  |  |  |  |  |  |  | 1 |  |  |  |
|  | *Trigona braueri* |  |  | 1 |  |  | 1 |  |  |  |  |  |  |  | 1 |  | 1 | 1 |  |  |
|  | *Trigona* sp.01 |  |  |  |  |  |  |  |  |  |  |  |  |  | 1 |  | 1 |  |  |  |
|  | *Trigona* sp.02 |  |  | 1 |  |  |  |  |  |  |  |  |  |  |  |  |  | 1 |  |  |
|  | *Trigona spinipes* |  |  | 1 | 1 | 1 |  |  | 1 | 1 |  |  |  |  | 1 |  | 1 | 1 | 1 | 1 |
| Butterfly | *Archaeoprepona demophoon* |  |  |  | 1 |  |  | 1 |  |  |  |  |  |  |  |  | 1 |  |  |  |
|  | *Blepolenis batea* |  |  | 1 |  | 1 |  | 1 | 1 | 1 |  |  |  |  | 1 |  | 1 | 1 | 1 |  |
|  | *Caligo arisbe* |  |  |  | 1 |  | 1 | 1 | 1 | 1 | 1 | 1 |  |  |  |  | 1 | 1 | 1 | 1 |
|  | *Callicore sorana* |  |  |  |  | 1 |  |  | 1 |  |  |  |  | 1 |  |  |  | 1 | 1 | 1 |
|  | *Carminda griseldis* |  |  |  |  | 1 | 1 | 1 |  |  |  |  |  |  |  |  | 1 |  | 1 | 1 |
|  | *Catonephele acontius* |  |  |  |  |  |  |  |  |  |  |  |  |  | 1 |  |  | 1 |  | 1 |
|  | *Catonephele numilia* |  |  |  |  |  |  | 1 |  |  |  |  |  |  |  |  |  |  | 1 | 1 |
|  | *Colobura dirce* |  |  |  |  |  |  |  |  |  |  |  |  | 1 |  |  |  |  | 1 | 1 |
|  | *Dasyophthalma rusina* |  | 1 |  | 1 |  |  |  | 1 |  |  |  |  |  | 1 |  |  | 1 |  | 1 |
|  | *Eryphanis automedon* |  |  |  | 1 |  |  |  |  | 1 |  |  |  |  |  |  | 1 |  |  |  |
|  | *Eryphanis reevesi* |  |  |  |  |  |  |  |  | 1 |  |  |  |  |  |  |  |  |  | 1 |
|  | *Euptychoides castrensis* |  |  | 1 |  |  |  | 1 |  |  |  |  |  |  |  |  |  |  | 1 | 1 |
|  | *Forsterinaria necys* | 1 |  | 1 |  |  |  |  |  | 1 | 1 |  |  |  |  |  | 1 |  | 1 | 1 |
|  | *Forsterinaria pronophila* |  |  |  |  |  |  |  |  |  |  |  |  |  | 1 |  |  |  | 1 | 1 |
|  | *Forsterinaria quantius* |  |  |  | 1 | 1 |  |  |  | 1 | 1 |  |  |  |  |  | 1 | 1 | 1 | 1 |
|  | *Godartiana muscosa* | 1 | 1 | 1 | 1 | 1 |  | 1 | 1 | 1 | 1 | 1 |  |  |  |  | 1 | 1 | 1 | 1 |
|  | *Hamadryas epinome* |  |  |  |  |  |  | 1 |  |  |  |  |  |  |  |  |  |  |  | 1 |
|  | *Hamadryas feronia* |  |  |  |  |  |  |  |  |  | 1 |  |  |  |  |  |  |  |  | 1 |
|  | *Hermeuptychia* sp.1 |  |  | 1 |  | 1 |  |  |  |  | 1 |  |  |  |  |  | 1 | 1 | 1 | 1 |
|  | *Memphis moruus* |  |  |  |  |  |  |  |  |  |  |  |  |  | 1 |  |  | 1 |  |  |
|  | *Moneuptychia itapeva* |  |  |  |  | 1 |  |  | 1 |  |  |  |  |  |  |  | 1 |  |  |  |
|  | *Moneuptychia soter* |  |  | 1 |  | 1 | 1 | 1 |  |  |  |  |  |  |  |  | 1 |  |  |  |
|  | *Myscelia orsis* |  |  |  |  |  |  |  |  |  |  |  |  |  | 1 |  |  | 1 |  |  |
|  | *Nica flavilla* |  |  |  |  |  |  |  |  |  | 1 |  |  |  |  |  |  |  | 1 | 1 |
|  | *Opoptera syme* |  |  |  |  |  |  | 1 | 1 | 1 | 1 |  |  |  |  |  | 1 | 1 |  |  |
|  | *Opsiphanes invirae* |  |  |  |  |  |  |  |  | 1 |  |  |  |  | 1 |  |  | 1 | 1 | 1 |
|  | *Paryphthimoides eous* |  |  | 1 |  | 1 |  |  |  |  |  |  |  |  |  |  | 1 |  | 1 | 1 |
|  | *Paryphthimoides phronius* |  |  | 1 |  | 1 |  |  |  |  |  |  |  |  |  |  |  |  | 1 | 1 |
|  | *Paryphthimoides poltys* |  |  |  |  | 1 |  | 1 | 1 |  |  | 1 |  | 1 |  |  | 1 | 1 | 1 | 1 |
|  | *Paryphthimoides* sp.1 |  |  |  |  | 1 |  |  |  |  | 1 |  |  |  |  |  |  | 1 | 1 | 1 |
|  | *Pharneuptychia pharnabazus* |  |  |  |  | 1 |  |  |  |  |  |  |  |  |  |  | 1 |  | 1 | 1 |
|  | *Pharneuptychia* sp.1 |  |  |  |  | 1 | 1 |  |  |  |  |  |  |  |  |  | 1 | 1 |  |  |
|  | *Praepedaliodes phanias* |  |  |  |  |  |  |  |  | 1 |  |  |  |  |  |  |  | 1 |  |  |
|  | *Taygetis virgilia* |  |  |  |  |  | 1 |  |  |  |  |  |  |  |  |  |  |  | 1 | 1 |
|  | *Yphthimoides angulares* | 1 |  | 1 |  | 1 |  | 1 |  |  |  | 1 |  |  |  |  | 1 | 1 |  |  |
|  | *Yphthimoides ochracea* |  |  | 1 |  |  |  |  | 1 | 1 | 1 |  |  |  |  |  | 1 | 1 |  |  |
|  | *Yphthimoides pacta* |  |  |  |  |  |  |  |  | 1 |  |  |  |  |  |  | 1 |  |  |  |
|  | *Yphthimoides renata* |  |  |  |  |  |  | 1 | 1 |  | 1 |  |  |  |  |  | 1 | 1 |  |  |
|  | *Yphthimoides straminea* | 1 |  | 1 | 1 | 1 |  | 1 | 1 | 1 | 1 | 1 | 1 | 1 |  |  | 1 | 1 | 1 | 1 |
|  | *Yphthimoides yphthima* |  |  |  |  |  |  |  |  |  | 1 |  |  |  |  |  |  |  |  | 1 |
|  | *Zaretys* sp.1 |  |  | 1 |  |  |  |  |  |  |  |  |  | 1 |  |  |  |  | 1 | 1 |
|  | *Zischkaia pronophila* |  |  |  |  |  |  |  |  |  |  |  |  |  | 1 |  | 1 |  |  |  |
| Dung beetle | *Anomiopus* sp.2 |  | 1 |  |  |  |  |  |  |  |  |  |  |  |  |  |  |  | 1 |  |
|  | *Ateuchus* aff. *ovalis* |  |  |  |  |  |  |  |  |  |  |  |  |  | 1 |  |  | 1 |  |  |
|  | *Ateuchus* sp.1 |  |  |  | 1 |  |  | 1 |  |  |  | 1 |  | 1 |  |  |  |  | 1 | 1 |
|  | *Ateuchus* sp.2 |  |  |  |  |  | 1 | 1 |  |  |  |  | 1 | 1 |  |  |  |  | 1 | 1 |
|  | *Ateuchus* sp.3 |  |  |  |  |  |  |  |  |  |  |  | 1 |  |  |  |  |  | 1 |  |
|  | *Ateuchus subquadratus* |  |  |  |  | 1 |  |  |  |  |  |  |  |  |  |  |  | 1 |  |  |
|  | *Canthidium* sp.2 |  |  |  |  |  |  | 1 |  |  |  |  | 1 |  |  |  | 1 |  | 1 |  |
|  | *Canthidium* sp.3 | 1 |  |  | 1 |  | 1 | 1 | 1 | 1 | 1 |  | 1 | 1 | 1 |  | 1 | 1 | 1 | 1 |
|  | *Canthidium* sp.4 | 1 |  | 1 |  |  | 1 |  | 1 |  | 1 |  |  |  | 1 |  | 1 | 1 | 1 | 1 |
|  | *Canthidium* sp.5 | 1 | 1 | 1 | 1 | 1 | 1 |  | 1 | 1 | 1 | 1 | 1 |  | 1 |  | 1 | 1 | 1 | 1 |
|  | *Canthidium* sp.6 |  |  |  |  |  | 1 |  |  |  |  | 1 |  |  |  |  |  | 1 |  |  |
|  | *Canthidium* sp.7 |  |  |  |  |  | 1 |  | 1 |  | 1 |  |  |  |  |  |  | 1 |  | 1 |
|  | *Canthidium* sp.12 |  |  |  |  |  |  |  |  |  | 1 |  |  |  |  |  |  |  |  | 1 |
|  | *Canthon* aff. *luctuosus* |  |  |  |  |  |  |  |  |  |  |  |  | 1 |  |  |  | 1 |  |  |
|  | *Canthon* aff. *simulans* |  |  |  |  |  |  |  |  |  | 1 |  |  |  |  |  |  |  |  | 1 |
|  | *Chalcocopris* *hesperus* | 1 |  |  |  |  |  |  | 1 | 1 |  |  | 1 |  |  |  | 1 | 1 | 1 | 1 |
|  | *Deltochilum* aff. *furcatum* |  | 1 |  |  |  |  |  |  |  |  |  |  |  |  |  |  |  |  | 1 |
|  | *Deltochilum* aff. *morbillosum* |  |  |  |  |  |  |  |  |  |  |  |  |  | 1 |  |  |  | 1 |  |
|  | *Deltochilum brasiliense* |  | 1 |  | 1 |  | 1 | 1 |  |  | 1 | 1 | 1 | 1 |  |  | 1 | 1 | 1 | 1 |
|  | *Deltochilum pseudoicarus* |  |  |  |  |  |  |  | 1 |  |  |  |  |  |  |  |  | 1 |  |  |
|  | *Deltochilum* sp.1 |  |  |  |  | 1 |  |  |  |  |  |  |  |  |  |  |  | 1 |  |  |
|  | *Deltochilum* sp.2 |  |  |  |  |  |  |  |  | 1 |  |  |  |  | 1 |  | 1 |  |  |  |
|  | *Dichotomius* aff. *zikani* |  |  |  |  |  | 1 | 1 |  |  |  |  | 1 |  |  |  | 1 |  | 1 | 1 |
|  | *Dichotomius affinis* | 1 | 1 |  | 1 | 1 | 1 |  |  |  |  |  |  |  |  |  | 1 | 1 | 1 | 1 |
|  | *Dichotomius bechynei* | 1 | 1 | 1 | 1 |  | 1 | 1 |  | 1 | 1 |  | 1 |  | 1 |  |  | 1 | 1 | 1 |
|  | *Dichotomius fissus* | 1 | 1 | 1 | 1 | 1 | 1 | 1 |  | 1 | 1 | 1 | 1 | 1 | 1 |  | 1 | 1 | 1 | 1 |
|  | *Dichotomius luctuosus* |  |  | 1 |  |  |  |  |  |  |  |  |  |  |  |  |  |  | 1 |  |
|  | *Dichotomius punctulatipennis* |  |  |  | 1 |  |  |  |  | 1 | 1 |  |  |  | 1 |  | 1 |  |  | 1 |
|  | *Dichotomius* sp.1 |  |  |  | 1 | 1 |  |  | 1 | 1 | 1 | 1 | 1 | 1 |  |  | 1 | 1 | 1 | 1 |
|  | *Dichotomius* sp.2 | 1 | 1 | 1 | 1 | 1 | 1 | 1 | 1 | 1 | 1 | 1 |  | 1 |  |  | 1 | 1 | 1 | 1 |
|  | *Dichotomius* sp.3 | 1 | 1 | 1 | 1 | 1 | 1 | 1 | 1 | 1 | 1 |  |  | 1 |  |  | 1 | 1 | 1 | 1 |
|  | *Dichotomius* sp.4 |  |  |  |  |  |  |  |  |  |  | 1 |  | 1 |  |  | 1 |  |  |  |
|  | *Dichotomius* sp.5 |  |  |  |  |  |  | 1 |  | 1 | 1 | 1 |  |  |  |  |  | 1 | 1 | 1 |
|  | *Dichotomius* sp.6 |  |  |  |  |  |  |  |  |  |  |  |  | 1 |  |  | 1 |  |  |  |
|  | *Dichotomius* sp.8 |  |  |  |  | 1 |  |  | 1 |  | 1 |  |  |  |  |  |  |  | 1 | 1 |
|  | *Dichotomius* sp.10 |  |  |  |  |  |  |  | 1 |  |  |  |  |  |  |  |  |  |  | 1 |
|  | *Dichotomius* sp.11 |  | 1 |  |  |  |  |  |  |  |  |  |  |  | 1 |  |  |  |  | 1 |
|  | *Eurysternus cyanescens* |  |  |  | 1 |  | 1 |  | 1 | 1 | 1 |  |  |  | 1 |  | 1 | 1 | 1 | 1 |
|  | *Eurysternus deplanatus* |  |  |  |  |  |  | 1 |  |  |  |  |  |  |  |  |  |  |  | 1 |
|  | *Eurysternus nigrovirens* |  |  |  | 1 |  |  |  | 1 |  |  |  |  |  |  |  | 1 | 1 | 1 | 1 |
|  | *Holocephalus eridanus* |  |  |  | 1 |  | 1 |  |  |  |  |  |  |  |  |  |  | 1 |  |  |
|  | *Ontherus azteca* |  |  |  | 1 |  |  |  |  |  |  |  |  |  |  |  |  |  | 1 | 1 |
|  | *Ontherus* sp.1 |  |  | 1 |  |  |  |  |  |  |  |  |  |  |  |  | 1 |  |  |  |
|  | *Onthophagus catharinensis* |  | 1 |  | 1 |  |  |  |  |  |  |  |  |  | 1 |  |  |  | 1 | 1 |
|  | *Onthophagus hircus* |  |  | 1 |  | 1 |  |  |  |  | 1 |  |  |  |  |  |  |  |  | 1 |
|  | *Onthophagus* sp.1 | 1 |  |  |  | 1 |  | 1 | 1 |  | 1 | 1 |  | 1 |  |  | 1 | 1 | 1 |  |
|  | *Paracanthon marinezae* |  |  |  | 1 |  |  | 1 |  | 1 | 1 | 1 |  |  |  |  | 1 | 1 | 1 | 1 |
|  | *Phanaeus dejeani* |  |  |  |  |  |  |  |  | 1 |  |  |  |  |  |  | 1 |  | 1 | 1 |
|  | *Phanaeus splendidulus* | 1 |  |  |  |  |  |  |  |  |  |  |  |  |  |  |  |  |  | 1 |
|  | *Sulcophanaeus menelas* |  |  | 1 |  | 1 |  |  | 1 |  |  |  |  |  |  |  | 1 |  | 1 | 1 |
|  | *Sylvicanthon foveiventris* | 1 | 1 | 1 | 1 |  |  |  |  | 1 |  |  |  |  | 1 |  | 1 | 1 | 1 | 1 |
|  | *Trichillum* sp.3 |  |  |  | 1 |  | 1 |  | 1 |  |  |  |  |  |  |  | 1 | 1 | 1 |  |
|  | *Uroxys aterrima* | 1 | 1 | 1 |  | 1 |  | 1 | 1 | 1 | 1 |  |  |  |  |  | 1 | 1 | 1 | 1 |
|  | *Uroxys* sp.1 | 1 | 1 | 1 | 1 | 1 | 1 | 1 | 1 | 1 | 1 | 1 | 1 | 1 | 1 |  | 1 | 1 | 1 | 1 |
|  | *Uroxys* sp.2 | 1 | 1 | 1 | 1 | 1 | 1 | 1 | 1 | 1 | 1 | 1 | 1 | 1 | 1 |  | 1 | 1 | 1 | 1 |
| Wasp | *Aelurus* sp.01 | 1 | 1 | 1 | 1 | 1 | 1 | 1 | 1 | 1 |  |  | 1 | 1 | 1 |  | 1 | 1 | 1 | 1 |
|  | *Aelurus* sp.02 |  |  | 1 |  |  |  |  | 1 | 1 |  |  |  | 1 |  |  | 1 | 1 |  |  |
|  | *Aelurus* sp.03 | 1 | 1 | 1 | 1 |  |  | 1 | 1 |  |  |  | 1 | 1 | 1 |  | 1 | 1 | 1 | 1 |
|  | *Agelaia multipicta* | 1 | 1 | 1 | 1 | 1 | 1 | 1 | 1 | 1 | 1 | 1 | 1 | 1 | 1 |  | 1 | 1 | 1 | 1 |
|  | *Agelaia myrmecophila* |  |  |  |  |  | 1 |  |  |  |  |  |  |  |  |  | 1 | 1 |  |  |
|  | *Ampulex* sp.01 |  |  |  |  |  |  |  |  | 1 |  | 1 |  | 1 |  |  | 1 | 1 |  |  |
|  | *Ampulex* sp.02 |  |  |  |  |  |  |  |  |  |  |  |  |  | 1 |  |  | 1 |  |  |
|  | Anthoboscinae sp.01 |  |  |  |  |  |  | 1 | 1 |  |  |  |  |  | 1 |  |  | 1 |  | 1 |
|  | Bethylinae sp.01 |  |  |  |  |  |  |  |  |  |  | 1 |  |  |  |  |  | 1 |  |  |
|  | Bothynostethini sp.01 |  |  |  |  |  |  |  |  |  |  |  |  | 1 | 1 |  | 1 | 1 |  |  |
|  | *Brachygastra mellifica* |  |  |  |  |  | 1 |  |  |  |  |  |  |  |  |  |  |  |  | 1 |
|  | *Caenochrysis* sp.01 |  |  |  |  | 1 |  |  |  |  |  |  |  |  |  |  |  | 1 |  |  |
|  | *Campsomeris* sp.01 |  |  | 1 | 1 |  |  | 1 |  |  | 1 | 1 |  | 1 |  |  | 1 | 1 |  |  |
|  | *Campsomeris* sp.02 |  | 1 |  | 1 |  | 1 | 1 |  |  |  |  | 1 | 1 | 1 |  | 1 | 1 | 1 | 1 |
|  | *Campsomeris* sp.03 |  |  |  |  |  |  | 1 |  | 1 | 1 | 1 | 1 |  |  |  |  | 1 |  | 1 |
|  | *Campsomeris* sp.04 |  |  | 1 |  |  |  |  |  |  |  |  |  |  |  |  |  | 1 |  |  |
|  | *Campsomeris* sp.05 |  |  |  |  |  | 1 |  |  |  |  |  |  |  |  |  |  |  |  | 1 |
|  | *Campsomeris* sp.08 |  | 1 |  |  |  |  |  | 1 |  |  | 1 |  |  |  |  |  |  | 1 | 1 |
|  | Crabronidae sp.36 |  |  |  |  |  | 1 |  |  |  |  |  |  |  |  |  |  |  |  | 1 |
|  | Crabronini sp.01 |  |  | 1 |  | 1 |  |  |  |  | 1 |  |  | 1 |  |  | 1 | 1 |  |  |
|  | Crabronini sp.02 |  |  |  |  |  |  | 1 | 1 |  |  |  | 1 |  | 1 |  | 1 | 1 |  |  |
|  | Crabronini sp.04 |  |  |  |  | 1 |  |  |  |  |  |  |  |  |  |  | 1 |  |  |  |
|  | *Didineis* sp.01 | 1 |  |  | 1 |  | 1 | 1 | 1 |  | 1 | 1 | 1 |  | 1 |  | 1 | 1 | 1 |  |
|  | *Dolichurus* sp.01 |  | 1 | 1 |  |  |  |  |  |  |  |  |  |  |  |  | 1 | 1 |  |  |
|  | *Dolichurus* sp.02 |  | 1 | 1 |  |  |  |  |  |  |  |  |  | 1 | 1 |  | 1 | 1 |  |  |
|  | Dryinidae sp.01 |  |  |  |  |  |  |  |  |  |  |  |  |  | 1 |  | 1 |  |  |  |
|  | *Dryinus* sp.01 |  |  |  |  |  |  |  |  |  |  |  |  |  | 1 |  | 1 | 1 |  |  |
|  | *Dryinus* sp.02 |  |  | 1 |  |  |  |  | 1 |  |  |  |  |  | 1 |  | 1 | 1 |  |  |
|  | Dryinus sp.04 |  |  | 1 |  |  |  |  |  |  |  |  |  |  | 1 |  | 1 | 1 |  |  |
|  | Ectemnius sp.01 |  |  |  |  | 1 |  |  |  | 1 |  |  |  |  |  |  | 1 | 1 |  |  |
|  | *Ephuta* sp.01 | 1 | 1 | 1 | 1 | 1 | 1 | 1 |  | 1 | 1 | 1 | 1 | 1 | 1 |  | 1 | 1 | 1 | 1 |
|  | *Ephuta* sp.02 |  |  |  |  |  |  |  |  | 1 |  |  |  |  | 1 |  | 1 | 1 |  |  |
|  | *Ephuta* sp.03 |  |  |  |  |  |  |  | 1 |  |  |  |  |  |  |  | 1 |  |  |  |
|  | *Ephuta* sp.04 |  |  |  |  | 1 |  | 1 | 1 | 1 | 1 | 1 | 1 |  |  |  |  |  | 1 | 1 |
|  | *Ephuta* sp.05 |  |  |  |  | 1 |  |  |  |  |  |  |  |  |  |  |  | 1 |  |  |
|  | Epyrinae sp.01 | 1 | 1 | 1 | 1 | 1 | 1 | 1 | 1 | 1 |  | 1 | 1 | 1 | 1 |  | 1 | 1 |  |  |
|  | Epyrinae sp.02 | 1 | 1 |  |  |  | 1 | 1 |  | 1 |  | 1 | 1 | 1 | 1 |  | 1 | 1 |  | 1 |
|  | Epyrinae sp.04 |  |  |  |  |  |  |  |  |  |  |  |  |  | 1 |  | 1 |  |  |  |
|  | Epyrinae sp.06 |  |  |  |  |  | 1 |  |  |  |  |  |  |  |  |  |  | 1 |  |  |
|  | *Eremnophila* sp.01 |  |  |  |  | 1 |  |  |  |  |  |  |  |  |  |  | 1 |  |  |  |
|  | *Eucerceris* sp.01 |  |  |  |  | 1 |  |  |  |  | 1 |  |  |  |  |  | 1 |  | 1 | 1 |
|  | *Eucerceris* sp.02 |  |  |  |  |  |  |  |  |  |  |  | 1 |  |  |  |  | 1 |  |  |
|  | Gastroserina sp.02 |  |  |  |  | 1 |  |  |  |  |  |  |  |  |  |  | 1 |  |  |  |
|  | *Ipsiura* sp.01 |  |  |  |  | 1 | 1 |  |  |  |  |  |  |  |  |  | 1 | 1 |  |  |
|  | *Liris* sp.01 |  | 1 |  |  | 1 | 1 | 1 |  |  |  | 1 | 1 |  | 1 |  | 1 | 1 |  |  |
|  | *Mischocyttarus rotundicollis* |  | 1 | 1 |  | 1 | 1 | 1 |  |  | 1 | 1 |  | 1 |  |  | 1 | 1 | 1 | 1 |
|  | Miscophini sp.01 |  |  |  |  |  |  | 1 |  |  |  |  | 1 |  |  |  | 1 | 1 |  |  |
|  | *Ornepetes* sp.01 | 1 |  |  |  |  |  |  |  |  |  |  |  |  |  |  |  |  |  | 1 |
|  | Pemphredonini sp.01 |  |  |  |  | 1 |  | 1 |  |  |  |  |  |  | 1 |  | 1 | 1 |  |  |
|  | Pemphredonini sp.02 |  |  |  | 1 |  |  |  |  |  |  |  |  |  | 1 |  |  | 1 |  |  |
|  | Pemphredonini sp.03 |  |  |  |  |  |  |  |  |  |  |  |  |  | 1 |  | 1 |  |  |  |
|  | Pemphredonini sp.04 |  |  |  |  |  |  |  |  |  |  |  |  |  | 1 |  | 1 |  |  |  |
|  | *Pleurochrysis* sp.01 |  |  |  |  |  |  |  |  |  |  |  | 1 |  |  |  |  | 1 |  |  |
|  | *Polistes versicolor* |  |  |  |  |  |  |  |  | 1 | 1 |  |  |  |  |  |  | 1 | 1 |  |
|  | *Polybia bifasciata* |  |  | 1 | 1 | 1 |  | 1 |  |  | 1 | 1 | 1 | 1 |  |  | 1 | 1 | 1 | 1 |
|  | *Polybia chrysothorax* |  |  |  |  |  |  |  |  |  |  |  |  | 1 |  |  |  |  |  | 1 |
|  | *Polybia fastidiosuscula* | 1 | 1 | 1 | 1 | 1 | 1 | 1 | 1 | 1 | 1 | 1 | 1 | 1 | 1 |  | 1 | 1 | 1 | 1 |
|  | *Polybia lugubris* |  |  |  | 1 |  |  | 1 |  |  |  |  |  |  |  |  | 1 |  | 1 |  |
|  | *Polybia occidentalis* |  |  |  | 1 |  |  |  |  |  | 1 |  |  |  |  |  |  | 1 |  |  |
|  | *Polybia sericea* |  |  |  |  | 1 |  | 1 | 1 |  |  | 1 | 1 |  |  |  | 1 | 1 | 1 | 1 |
|  | *Polybia* sp. |  |  |  |  | 1 |  |  |  |  |  |  |  |  |  |  | 1 |  |  |  |
|  | Pompilidae sp.02 | 1 | 1 |  | 1 |  | 1 | 1 | 1 | 1 | 1 | 1 | 1 |  |  |  | 1 | 1 | 1 | 1 |
|  | Pompilidae sp.04 |  |  |  |  |  | 1 | 1 | 1 | 1 | 1 |  | 1 |  |  |  | 1 | 1 |  |  |
|  | Pompilidae sp.06 |  |  |  |  |  |  |  | 1 |  |  |  |  |  | 1 |  |  | 1 |  |  |
|  | Pompilidae sp.07 |  |  |  |  |  |  |  |  |  |  | 1 |  |  |  |  | 1 |  |  |  |
|  | Pompilidae sp.09 |  |  |  |  |  |  |  |  | 1 |  |  |  |  |  |  | 1 |  |  |  |
|  | Pompilidae sp.10 |  |  |  |  |  |  |  |  | 1 |  |  |  |  |  |  |  | 1 |  |  |
|  | Pompilidae sp.11 |  |  |  |  |  |  |  | 1 | 1 |  |  |  |  |  |  | 1 | 1 |  |  |
|  | Pompilidae sp.13 |  |  |  |  |  | 1 | 1 |  |  |  |  |  | 1 |  |  | 1 | 1 |  |  |
|  | Pompilidae sp.14 |  | 1 |  | 1 |  |  |  | 1 | 1 |  |  |  |  |  |  | 1 | 1 |  |  |
|  | Pompilidae sp.15 |  |  |  |  |  | 1 | 1 |  |  | 1 | 1 | 1 | 1 |  |  | 1 | 1 | 1 | 1 |
|  | Pompilidae sp.16 |  |  |  |  | 1 | 1 | 1 |  |  |  |  | 1 | 1 |  |  | 1 | 1 |  |  |
|  | Pompilidae sp.18 |  |  |  | 1 |  | 1 |  |  | 1 | 1 |  | 1 | 1 | 1 |  | 1 | 1 |  |  |
|  | Pompilidae sp.19 | 1 | 1 | 1 | 1 | 1 |  | 1 | 1 | 1 | 1 | 1 | 1 | 1 |  |  | 1 | 1 | 1 | 1 |
|  | Pompilidae sp.20 |  | 1 | 1 |  | 1 | 1 | 1 |  |  |  | 1 | 1 |  |  |  | 1 | 1 |  |  |
|  | Pompilidae sp.21 |  |  |  |  |  |  | 1 |  |  |  |  |  |  |  |  | 1 |  |  |  |
|  | Pompilidae sp.23 |  |  |  | 1 |  |  |  |  |  |  | 1 | 1 |  |  |  | 1 | 1 |  |  |
|  | Pompilidae sp.24 |  |  |  |  |  | 1 |  |  | 1 | 1 | 1 |  |  |  |  | 1 | 1 | 1 |  |
|  | Pompilidae sp.25 |  |  |  |  |  |  |  |  |  |  |  |  |  | 1 |  | 1 |  |  |  |
|  | Pompilidae sp.26 |  | 1 |  |  |  | 1 |  |  |  |  | 1 | 1 |  |  |  |  | 1 |  |  |
|  | Pompilidae sp.27 |  |  |  |  | 1 | 1 | 1 |  |  | 1 | 1 | 1 |  | 1 |  | 1 | 1 |  |  |
|  | Pompilidae sp.28 |  | 1 | 1 |  |  | 1 | 1 |  |  |  | 1 |  |  |  |  | 1 | 1 |  |  |
|  | Pompilidae sp.29 |  | 1 |  |  |  |  |  |  |  |  |  |  |  |  |  |  | 1 |  |  |
|  | Pompilidae sp.30 |  |  |  |  | 1 |  |  |  |  |  |  |  |  |  |  |  | 1 |  |  |
|  | Pompilidae sp.31 |  |  |  |  | 1 |  | 1 |  |  |  |  |  |  |  |  |  | 1 |  |  |
|  | Pompilidae sp.32 |  |  |  |  |  | 1 |  |  |  |  |  | 1 |  |  |  | 1 |  |  |  |
|  | Pompilidae sp.33 |  |  |  |  |  | 1 |  |  |  |  |  | 1 |  |  |  |  | 1 |  |  |
|  | Pompilidae sp.34 |  |  |  |  |  |  |  |  |  |  | 1 |  |  |  |  |  | 1 |  |  |
|  | Pompilidae sp.35 |  |  |  |  | 1 |  |  |  |  |  |  |  |  |  |  |  | 1 |  |  |
|  | Pompilidae sp.36 |  |  |  |  |  |  | 1 |  |  |  |  |  |  |  |  |  | 1 |  |  |
|  | Pompilidae sp.38 |  |  |  |  | 1 |  |  |  |  |  |  |  |  |  |  | 1 |  |  |  |
|  | Pompilidae sp.39 |  |  |  |  | 1 |  |  |  |  |  |  |  |  |  |  | 1 |  |  |  |
|  | Pompilidae sp.40 |  |  |  |  |  |  |  |  |  |  |  | 1 |  |  |  |  | 1 |  |  |
|  | Pompilidae sp.41 |  |  |  |  |  |  |  |  |  |  |  | 1 |  |  |  |  | 1 |  |  |
|  | Pristocerinae sp.01 |  |  |  | 1 |  |  |  | 1 |  |  |  | 1 |  | 1 |  | 1 | 1 |  |  |
|  | Pristocerinae sp.02 |  |  |  | 1 |  | 1 | 1 |  |  |  |  |  |  | 1 |  |  | 1 |  |  |
|  | Pristocerinae sp.03 |  |  |  |  |  |  |  |  | 1 |  |  |  |  | 1 |  | 1 | 1 |  |  |
|  | Pristocerinae sp.04 |  | 1 |  | 1 | 1 |  |  | 1 |  | 1 |  |  | 1 |  |  | 1 | 1 |  |  |
|  | Pristocerinae sp.05 | 1 | 1 | 1 | 1 | 1 | 1 | 1 | 1 | 1 | 1 | 1 | 1 | 1 | 1 |  | 1 | 1 |  |  |
|  | Pristocerinae sp.06 | 1 | 1 | 1 | 1 | 1 | 1 | 1 | 1 |  | 1 | 1 | 1 | 1 | 1 |  | 1 | 1 | 1 |  |
|  | Pristocerinae sp.07 |  | 1 |  |  |  | 1 |  |  |  |  | 1 |  | 1 |  |  | 1 |  |  |  |
|  | Pristocerinae sp.08 |  |  |  |  |  |  | 1 |  |  |  |  |  | 1 |  |  | 1 | 1 |  |  |
|  | Pristocerinae sp.09 |  |  |  |  |  |  |  | 1 |  |  |  |  |  | 1 |  | 1 |  |  |  |
|  | Pristocerinae sp.10 |  |  | 1 |  |  |  |  |  | 1 |  |  |  |  |  |  | 1 | 1 |  |  |
|  | Pristocerinae sp.11 |  | 1 |  | 1 |  |  |  | 1 |  | 1 |  |  |  | 1 |  | 1 | 1 |  |  |
|  | Pristocerinae sp.12 |  | 1 |  |  |  |  |  |  |  |  |  |  |  |  |  |  | 1 |  |  |
|  | Pristocerinae sp.14 |  |  |  |  |  |  |  | 1 |  |  |  |  |  |  |  | 1 |  |  |  |
|  | *Pterombrus* sp.01 |  | 1 |  | 1 |  | 1 |  | 1 |  |  |  |  |  | 1 |  | 1 | 1 |  |  |
|  | Scotaenini sp.02 |  |  |  |  |  |  |  |  |  |  |  |  |  | 1 |  | 1 | 1 |  |  |
|  | Scotaenini sp.03 |  |  |  |  |  |  |  |  |  |  |  |  |  | 1 |  |  | 1 |  |  |
|  | Sphaeropthalmini sp.01 |  |  |  |  |  | 1 |  |  |  |  |  |  |  |  |  |  | 1 |  |  |
|  | Sphaeropthalmini sp.02 |  |  |  |  | 1 |  |  |  |  |  |  |  | 1 |  |  | 1 | 1 |  |  |
|  | Sphaeropthalmini sp.05 | 1 |  |  |  |  | 1 |  |  | 1 |  |  |  |  |  |  |  |  | 1 |  |
|  | *Spilomena* sp.01 |  |  |  |  | 1 |  |  |  |  |  | 1 | 1 | 1 |  |  | 1 | 1 |  |  |
|  | *Spilomena* sp.02 |  |  |  |  |  |  |  |  |  |  |  |  |  | 1 |  | 1 | 1 |  |  |
|  | *Spilomena* sp.03 |  |  |  |  | 1 |  |  |  |  |  |  |  |  | 1 |  |  | 1 |  | 1 |
|  | *Thaumatodryinus* sp.01 |  |  |  |  | 1 |  |  |  |  |  |  |  |  |  |  | 1 |  |  |  |
|  | *Thaumatodryinus* sp.02 |  |  |  |  |  |  |  |  |  |  |  |  |  | 1 |  | 1 |  |  |  |
|  | *Timulla* sp.01 |  |  |  |  | 1 |  |  |  |  |  |  |  |  |  |  |  | 1 |  |  |
|  | *Tiphia* sp.01 | 1 |  | 1 |  | 1 | 1 | 1 | 1 | 1 | 1 | 1 | 1 | 1 | 1 |  | 1 | 1 | 1 |  |
|  | *Tiphia* sp.02 |  |  |  |  | 1 |  | 1 | 1 |  | 1 | 1 | 1 | 1 | 1 |  | 1 | 1 |  |  |
|  | *Tiphia* sp.03 |  |  | 1 |  |  |  | 1 |  |  |  | 1 |  |  | 1 |  | 1 |  | 1 | 1 |
|  | Trypoxylini sp.01 | 1 |  |  |  |  | 1 | 1 | 1 |  |  |  | 1 |  | 1 |  | 1 | 1 |  | 1 |
|  | Trypoxylini sp.02 |  |  |  |  |  |  |  | 1 | 1 |  | 1 |  |  | 1 |  | 1 | 1 |  |  |
|  | Trypoxylini sp.03 |  |  | 1 | 1 |  |  |  |  |  |  |  | 1 |  | 1 |  | 1 | 1 |  |  |
|  | Trypoxylini sp.04 |  |  | 1 |  | 1 | 1 | 1 |  | 1 |  | 1 |  |  |  |  |  | 1 | 1 |  |
|  | Trypoxylini sp.05 |  |  |  |  | 1 | 1 |  |  |  |  |  |  |  |  |  |  | 1 |  |  |
|  | Trypoxylini sp.06 |  | 1 |  |  |  |  |  | 1 |  |  |  |  |  |  |  |  | 1 |  |  |
|  | *Trypoxylon (Trypargilum) lactitarse* |  |  |  |  |  |  |  |  |  |  |  | 1 |  |  |  | 1 |  |  |  |
|  | *Xystromutilla* sp.01 |  |  |  |  | 1 | 1 |  |  |  |  |  |  |  | 1 |  | 1 | 1 |  |  |
|  | *Xystromutilla* sp.02 |  | 1 |  |  | 1 |  |  | 1 |  |  |  |  |  |  |  | 1 |  | 1 |  |
| **Number of species** | | **70** | **90** | **96** | **96** | **125** | **104** | **107** | **113** | **109** | **103** | **98** | **99** | **100** | **125** |  | **225** | **247** | **154** | **150** |

**Figure S1.** Images of some of the forest islands (a-d). We call continuous forest the much larger patch of forest located in the northeast of the map, which extends over a wider area and belongs to the Atlantic Forest domain. Photos in panels a-d by Lucas Perillo.

**
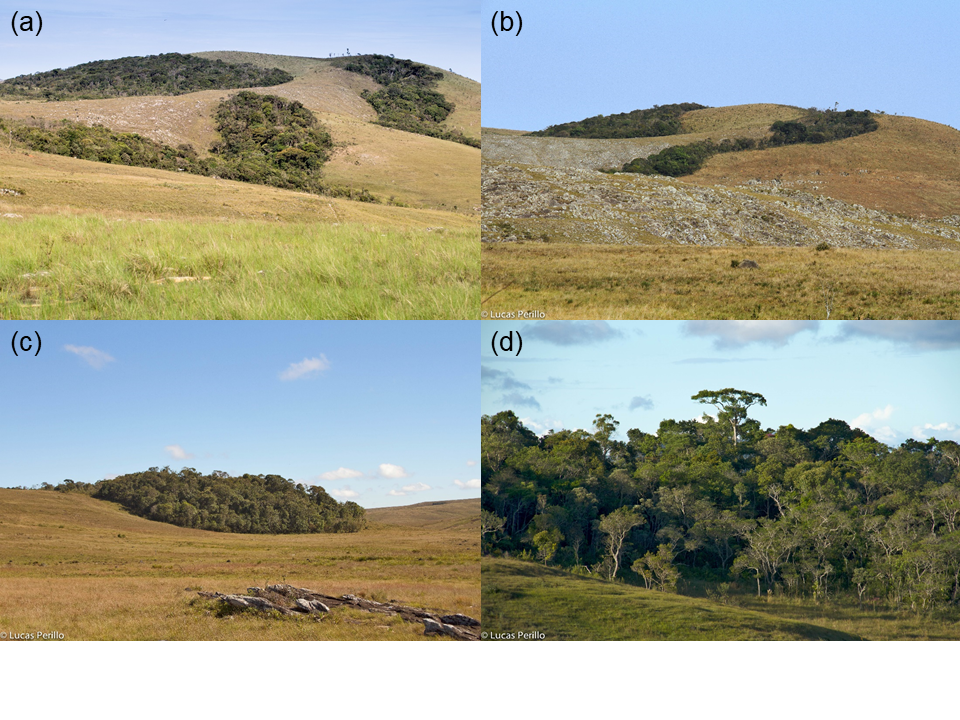
**

**Figure S2.** Rarefaction-extrapolation accumulation curves of richness and frequency of occurrence of a-b) ants, and richness and abundance of c-d) bees, e-f) butterflies, g-h) dung beetles, and i-j) wasps over the years. Observed and extrapolated values for a) antes sampled in 2014 (red), 2015 (green), 2018 (blue) and 2020 (purple); b) ants sampled in all years; c) bees sampled in 2014 (red), 2015 (green), 2018 (blue) and 2020 (purple); d) bees sampled in all years e) butterflies sampled in 2014 (red), 2015 (green), 2018 (blue) and 2020 (purple); f) butterflies sampled in all years g) dung beetles sampled in 2014 (red), 2015 (green), 2018 (blue) and 2020 (purple); h) dung beetles sampled in all years; i) wasps sampled in 2014 (red), 2015 (green), 2018 (blue) and 2020 (purple); j) wasps samples in all years.

**
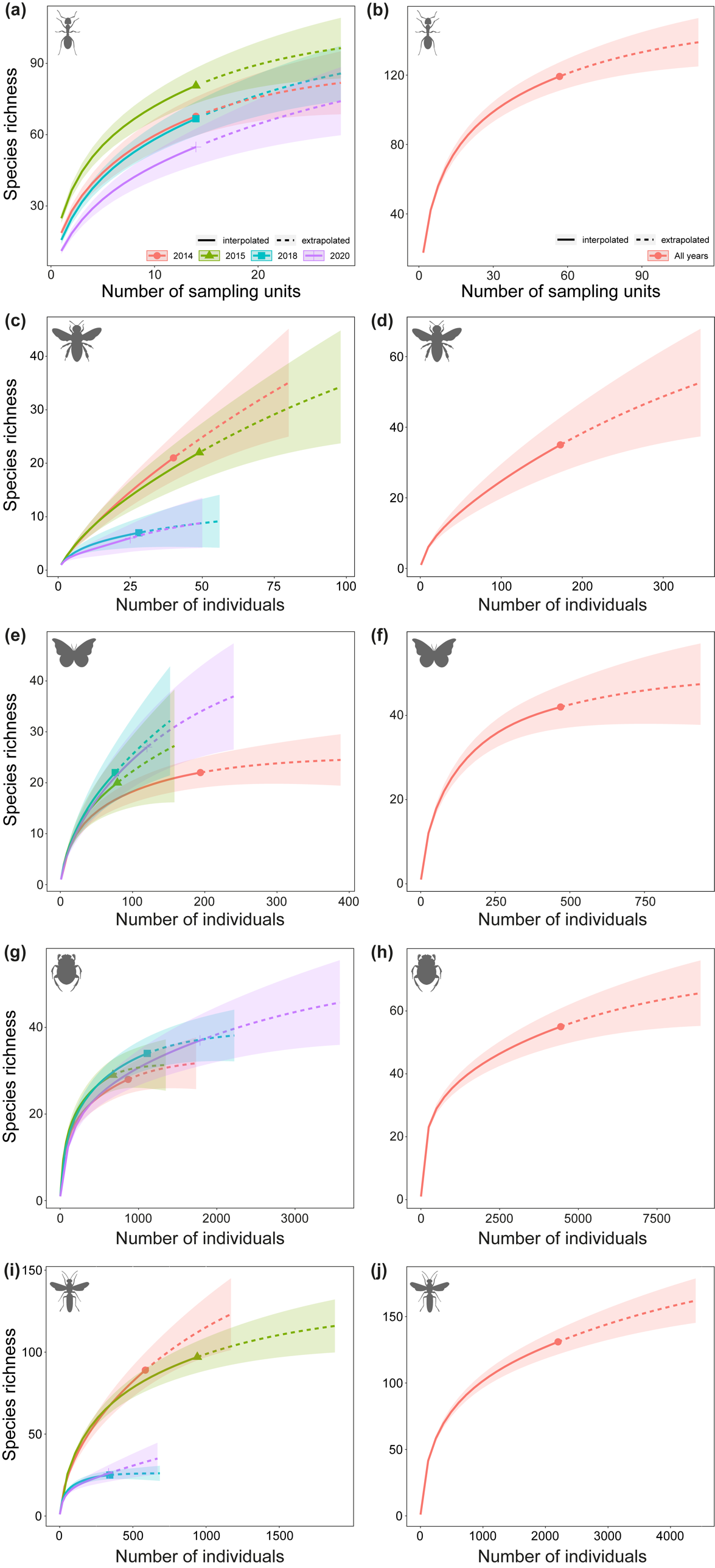
**
